# Genome-Wide Association Study for Serum Omega-3 and Omega-6 Polyunsaturated Fatty Acids: Exploratory Analysis of the Sex-Specific Effects and Dietary Modulation in Mediterranean Subjects with Metabolic Syndrome

**DOI:** 10.3390/nu12020310

**Published:** 2020-01-24

**Authors:** Oscar Coltell, Jose V. Sorlí, Eva M. Asensio, Rocío Barragán, José I. González, Ignacio M. Giménez-Alba, Vicente Zanón-Moreno, Ramon Estruch, Judith B. Ramírez-Sabio, Eva C. Pascual, Carolina Ortega-Azorín, Jose M. Ordovas, Dolores Corella

**Affiliations:** 1 Department of Computer Languages and Systems, Universitat Jaume I, 12071 Castellón, Spain; oscar.coltell@uji.es; 2 CIBER Fisiopatología de la Obesidad y Nutrición, Instituto de Salud Carlos III, 28029 Madrid, Spain; jose.sorli@uv.es (J.V.S.); eva.m.asensio@uv.es (E.M.A.); rocio.barragan@uv.es (R.B.); Ignacio.Glez-Arraez@uv.es (J.I.G.); nachoga16@gmail.com (I.M.G.-A.); restruch@clinic.cat (R.E.); Carolina.Ortega@uv.es (C.O.-A.); 3 Department of Preventive Medicine and Public Health, School of Medicine, University of Valencia, 46010 Valencia, Spain; pascaseva89@gmail.com; 4 Area of Health Sciences, Valencian International University, 46002 Valencia, Spain; vczanon@universidadviu.com; 5 Red Temática de Investigación Cooperativa en Patología Ocular (OFTARED), Instituto de Salud Carlos III, 28029 Madrid, Spain; 6 Ophthalmology Research Unit “Santiago Grisolia”, Dr. Peset University Hospital, 46017 Valencia, Spain; 7 Department of Internal Medicine, Hospital Clinic, Institut d’Investigació Biomèdica August Pi i Sunyer (IDIBAPS), University of Barcelona, 08036 Barcelona, Spain; 8 Oncology Department, Sagunto Hospital, 46500 Sagunto, Spain; jbramire@uv.es; 9 Assisted Reproduction Unit of the University Hospital of Valencia, 46010 Valencia, Spain; 10 Nutrition and Genomics Laboratory, JM-USDA Human Nutrition Research Center on Aging at Tufts University, Boston, MA 02111 USA; jose.ordovas@tufts.edu; 11 Department of Cardiovascular Epidemiology and Population Genetics, Centro Nacional de Investigaciones Cardiovasculares (CNIC), 28029 Madrid, Spain; 12 IMDEA Alimentación, 28049 Madrid, Spain

**Keywords:** polyunsaturated fatty acids, omega-3, omega-6, genetics, genome-wide association study, sex, Mediterranean population, heterogeneity, polymorphisms, metabolic syndrome

## Abstract

Many early studies presented beneficial effects of polyunsaturated fatty acids (PUFA) on cardiovascular risk factors and disease. However, results from recent meta-analyses indicate that this effect would be very low or nil. One of the factors that may contribute to the inconsistency of the results is that, in most studies, genetic factors have not been taken into consideration. It is known that fatty acid desaturase (*FADS*) gene cluster in chromosome 11 is a very important determinant of plasma PUFA, and that the prevalence of the single nucleotide polymorphisms (SNPs) varies greatly between populations and may constitute a bias in meta-analyses. Previous genome-wide association studies (GWAS) have been carried out in other populations and none of them have investigated sex and Mediterranean dietary pattern interactions at the genome-wide level. Our aims were to undertake a GWAS to discover the genes most associated with serum PUFA concentrations (omega-3, omega-6, and some fatty acids) in a scarcely studied Mediterranean population with metabolic syndrome, and to explore sex and adherence to Mediterranean diet (MedDiet) interactions at the genome-wide level. Serum PUFA were determined by NMR spectroscopy. We found strong robust associations between various SNPs in the *FADS* cluster and omega-3 concentrations (top-ranked in the adjusted model: *FADS1*-rs174547, *p* = 3.34 × 10^−14^; *FADS1*-rs174550, *p* = 5.35 × 10^−14^; *FADS2*-rs1535, *p* = 5.85 × 10^−14^; *FADS1*-rs174546, *p* = 6.72 × 10^−14^; *FADS2*-rs174546, *p* = 9.75 × 10^−14^; *FADS2*-rs174576, *p* = 1.17 × 10^−13^; *FADS2*-rs174577, *p* = 1.12 × 10^−12^, among others). We also detected a genome-wide significant association with other genes in chromosome 11: *MYRF* (myelin regulatory factor)-rs174535, *p* = 1.49 × 10^−12^; *TMEM258* (transmembrane protein 258)-rs102275, *p* = 2.43 × 10^−12^; *FEN1* (flap structure-specific endonuclease 1)-rs174538, *p* = 1.96 × 10^−11^). Similar genome-wide statistically significant results were found for docosahexaenoic fatty acid (DHA). However, no such associations were detected for omega-6 PUFAs or linoleic acid (LA). For total PUFA, we observed a consistent gene*sex interaction with the *DNTTIP2* (deoxynucleotidyl transferase terminal interacting protein 2)-rs3747965 *p* = 1.36 × 10^−8^. For adherence to MedDiet, we obtained a relevant interaction with the *ME1* (malic enzyme 1) gene (a gene strongly regulated by fat) in determining serum omega-3. The top-ranked SNP for this interaction was *ME1*-rs3798890 (*p* = 2.15 × 10^−7^). In the regional-wide association study, specifically focused on the *FADS1*/*FASD2*/*FADS3* and *ELOVL* (fatty acid elongase) 2/*ELOVL* 5 regions, we detected several statistically significant associations at *p* < 0.05. In conclusion, our results confirm a robust role of the *FADS* cluster on serum PUFA in this population, but the associations vary depending on the PUFA. Moreover, the detection of some sex and diet interactions underlines the need for these associations/interactions to be studied in all specific populations so as to better understand the complex metabolism of PUFA.

## 1. Introduction

Polyunsaturated fatty acids (PUFA) are fatty acids having more than one double bond on their carbon chain. Depending on the position of the first double bond close to the methyl end, they are classified as omega-6 or omega-3. These 2 families each encompass fatty acids with varying carbon chain length and degree of unsaturation. Supplemental Figure S1 shows PUFA biosynthesis, detailing the omega-6 and omega-3 pathways and the names of the main PUFA, as well as the elongation and the desaturation activities [1,2,3]. Briefly, the omega-6 PUFA linoleic acid (LA) and the omega-3 PUFA alpha-linolenic acid (ALA) from the diet are the key substrates that enter the pathways leading to the synthesis of the main long-chain PUFA. Long chain PUFA can be endogenously derived from (LA) or (ALA) by a consecutive series of desaturations (involving the Delta-6 desaturase encoded by the *FADS2* (fatty acid desaturase 2) gene and the Delta-5 desaturase, encoded by the *FADS1* (fatty acid desaturase 1) gene and elongation steps, as well as oxidation. Among the seven elongases, *ELOVL2* (elongation of very long chain fatty acids 2) and *ELOVL5* elongation of very long chain fatty acids 5) are PUFA-specific [4]. Docosahexaenoic acid (DHA) is a long-chain, highly unsaturated omega-3 fatty acid, synthesized from the ALA in this process. Both dietary and serum/plasma PUFA are a complex mix of different fatty acids. Circulating levels are determined by intake and by the biosynthesis pathways [5]. Omega-3 PUFA mainly exists in fish, marine oils, and other marine products, as well as in several plant sources [6,7,8,9]. Vegetable oils, nuts, seeds, meat, poultry, and cereal-based products are important sources of omega-6 PUFA [6,7,8,9].

Initial studies, both in humans and in animal models, showed several protective effects (hypotriglyceridemic, hypocholesterolemic, antithrombotic, anti-inflammatory, anti-arrhythmic, anti-hypertensive, improving vascular function and arterial compliance, etc.) of PUFA intake on cardiovascular risk [10,11,12,13,14,15,16]. This PUFA protective effect has been the focus of several dietary and pharmacological studies with PUFA on diverse cardiovascular risk factors as well as cardiovascular diseases for primary and secondary prevention. A detailed analysis of the findings of such studies is beyond the aim of the present work. There are many excellent reviews that cover these results [17,18,19,20,21,22,23,24,25,26,27,28,29]. However, the association between PUFA and cardiovascular risk factors and diseases has been surrounded by controversy [30,31,32,33,34,35] and the results of recent meta-analyses have been inconsistent [36,37,38,39,40,41].

Abdelhamid et al. [36] meta-analyzed 49 randomized, controlled trials (RCTs), examining the effects of total PUFA intake (including both diet or supplements) on lipids, adiposity, cardiovascular disease, and all-cause mortality. These authors concluded that increasing total PUFA intake probably reduces the risk of cardiovascular disease events slightly, but has little or no effect on all-cause or cardiovascular disease mortality [36]. Nevertheless, it is known that PUFA are a mix of different fatty acids. Early studies have already suggested that there could be differential effects of omega-6 and omega-3 PUFA on cardiovascular risk [42,43,44]. In general, it has been accepted that omega-3 PUFA helps decrease inflammation, whereas some omega-6 PUFA could promote inflammation [45,46,47,48,49]. The concept of a healthy omega-6 to omega-3 PUFA ratio in the diet, therefore, has been suggested [50,51]. Initially, the dietary content of omega-6 and omega-3 in the diet was well balanced, and a ratio close to 1:1 has been estimated for hunter–gatherer populations [52]. However, presently, Western diets are unbalanced worldwide due to an increase of dietary omega-6 and a decrease in omega-3. This results in a progressive shift in the omega-6/omega-3 ratio from 4:1 to >10:1 [53,54]. Although the optimal ratio has to be determined, an increased ratio may be harmful to human health and it has been related to metabolic dysfunction and higher cardiovascular risk in animal models and in human studies [48,49,50,51,52,53,54,55,56,57,58,59].

Nevertheless, recent studies suggest that omega-6 are not as harmful as initially thought [52,53]. Therefore, whether omega-6 and omega-3 PUFA oppositely contribute to a decrease in cardiovascular risk still remains debated. In a recent and comprehensive meta-analyses on the effects of omega-3 [37] and omega-6 PUFA [38] on cardiovascular risk, it has been pointed out that high quality evidence levels indicate that omega-3 PUFA has little or no effect on mortality or cardiovascular health. Likewise, the results indicated that increasing omega-6 PUFA only reduced myocardial infarction incidence but no other cardiovascular outcomes [39]. Similarly, Brown et al. [41] in an extensive meta-analysis including 83 RCTs analyzing the effect of PUFA for the prevention and treatment of type-2 diabetes, did not find any evidence that the omega-6/omega-3 ratio is important for diabetes or glucose metabolism.

Nonetheless, it has been claimed that the lack of conclusive evidence obtained in the meta-analyses may be due to several biases, including not only the poor quality of some of the meta-analyzed trials, but also other relevant factors such as the unmeasured role of genetic polymorphisms influencing the effect of dietary PUFA [1]. The potential role of *FADS* genetic polymorphism in determining the effects of dietary and pharmacological PUFA on several cardiovascular risk factors and outcomes, have been investigated by several studies [1,60,61,62,63,64,65]. Among them, a post-hoc analysis from the OMEGA-REMODEL RCT, investigating the effect of high-dose of omega-3 PUFA post-acute myocardial infarction [65] reported that the *FADS2* genotype can predict the beneficial effects of the omega-3. Another issue in these meta-analyses is the lack of focus on potential sex-specific differences, as well as overall dietary pattern modulating PUFA interventions. Regarding sex, several human studies as well as research in animal models [66,67,68,69,70,71,72,73] have reported some differences between males and females on analyzing the PUFA effects on diverse cardiovascular risk phenotypes. However, more specific research remains to be done in order to better understand the potential differences. Likewise, the effect of the concomitant dietary pattern (Mediterranean diet, Nordic diet, Western diet, Chinese diet, etc.) on the PUFA effects (diet or supplements) on cardiovascular phenotypes in different populations has been subject to increased attention in recent years [74,75,76,77,78]. However, more research is needed to better understand the dietary pattern influence.

All these factors should be considered in any new RCTs to be conducted in the field, as well as in the corresponding meta-analyses. In terms of the genetic factors to be analyzed in novel studies related to PUFA, although several genome-wide association studies (GWASs) for the different fatty acid concentrations in numerous tissues have been carried out in several populations, mainly of European ancestry [79,80,81,82,83,84,85,86], the percentage of variability explained by the polymorphisms discovered is still low for the different PUFA. Thus, additional research remains to be done to discover more PUFA-related genes. Despite the strong associations at the GWAS level between the *FADS1/2* locus in chromosome 11, and some fatty acids observed in all populations analyzed [79,80,81,82,83,84,85,86], some population-specific associations with other genetic variants and genes (i.e., *TRIM58*, *MCM6*, *NTAN1*/*PDXDC1*, *PKD2L1*, *PCOLCE2*, *AGPAT4*, *HBS1L*/*MYB*, *LPCAT3*) have also been described [79,81,82,84,86]. Specifically, in the GWAS carried out in Singaporean Chinese population [84] to test the homogeneity of previous findings in European ancestry populations, the authors found robust consistency for the main associations, but reported some heterogeneity for some loci (they were unable to detect the significant associations with *ELOVL2* single nucleotide polymorphisms (SNPs), and the NTAN1/PDXDC1 for the corresponding PUFA). Moreover, in another GWAS undertaken in Chinese participants, followed by a trans-ethnic meta-analysis including European ancestry participants [86], confirmed previously reported loci in the different ethnicities and also reported some specific associations in Chinese participants. In this sense, several studies have warned of the importance of knowing the most relevant specific variants in each population in order to minimize bias, since the results SNPs identified in GWAS of specific populations, cannot be directly extrapolated to other populations [87,88,89]. In view of that, the vast majority of GWASs for PUFA levels have been carried out in populations participating in the CHARGE (Cohorts for Heart and Aging Research in Genomic Epidemiology) Consortium [79,80,81,82,83], in Asian populations [84,86] and in Greenlandic Inuit subjects [85]. Although the CHARGE Consortium has analyzed one Italian population [79], no GWAS for serum PUFA has been carried out in Spain. Previous genetic analysis in this population has reported population-specific associations for some traits [90].

In general, GWASs for PUFA [79,80,81,82,83,84,85,86] have focused on serum/plasma or erythrocyte fatty acid levels. In these GWASs [79,80,81,82,83,84,85,86], a strong statistically significant signal has been consistently detected at the candidate region of the *FADS1*/*FADS2* cluster, previously identified in initial studies [91,92,93,94]. *FADS3* is the enigmatic third member of the *FADS* cluster [4]. It is important to bear in mind that the prevalence of the main SNPs in the *FADS* genes greatly varies among populations [95,96,97]. This reflects an evolutionary advantage of SNPs, enabling an active PUFA synthesis in response to drastic dietary differences [95,96,97,98]. Likewise, it has been suggested that there might be stronger selection pressure on the *FADS1*/*FASD2* block in southern Europeans [95]. In Spain, the adherence to the Mediterranean diet pattern [99] is still relatively high in older subjects, but is decreasing in young people [100,101]. Although some studies specifically carried out in the Spanish Mediterranean population, have analyzed the association between several *FADS* and *ELOVL* candidate gene polymorphisms and PUFA concentrations [102,103,104,105,106], no study has investigated such associations at the GWAS level.

Therefore, the potential genetic differences in the Mediterranean population, the urgent need for greater diversity in genetic analysis, and the fact that in previous GWASs, sex-specific differences as well as dietary interactions have scarcely been analyzed, prompted us to carry out this study with the following aims: (1) To investigate, at the genome-wide level, the genetic variants most associated with serum PUFA concentrations (focusing on omega-3 and omega-6 PUFA, and also examining DHA and LA) in a Mediterranean population with metabolic syndrome; (2) To analyze, at the region-wide (RWAS) level, the associations between the SNPs in the selected candidate regions and serum PUFA concentrations in this population; and (3) To explore the modulating influence of sex and adherence to Mediterranean diet (as a whole dietary pattern) on these associations.

## 2. Materials and Methods

### 2.1. Study Design and Participants

We analyzed participants in the PREDIMED Plus-Valencia study [105]. The University of Valencia (located in the eastern Mediterranean coast), is one of the field centers of the multi-center PREDIMED Plus study, an ongoing primary prevention trial conducted in Spain [106]. A detailed description of the trial is available at http://predimedplus.com/. This trial was registered at https://doi.org/10.1186/ISRCTN89898870. Participants in the Valencia field center were recruited from several primary care health facilities. These participants were community-dwelling adults (men, 55–75 years; women, 60–75 years) with body-mass index (BMI) ranging from 27 to 40 kg/m^2^ and having metabolic syndrome [106]. In this recruitment center, the total number of randomized participants included in the PREDIMED Plus trial was 465. In the present work, we undertake the cross-sectional analyses of an ancillary project including all the participants in our center with complete data on serum PUFA concentrations at baseline and genome-wide genotyping, in addition to the other variables for adjustments (n = 426). The analyzed subjects did not differ significantly from our entire sample concerning the main variables. The Institutional Review Board of the Valencia University approved the study protocol (ethical approval code H1373255532771), and all participants provided written informed consent.

### 2.2. Baseline Clinical, Anthropometric, Biochemical, and Lifestyle Variables

In the baseline examination, we assessed socio-demographic variables, clinical and cardiovascular risk factors, and lifestyle variables by validated questionnaires as previously reported [106]. Anthropometric variables and blood pressure were determined by trained staff following the PREDIMED Plus operations protocol [107]. Weight and height were measured with calibrated scales and a wall-mounted stadiometer, respectively. BMI was calculated as the weight in kilograms divided by the height in meters squared. Waist circumference was measured midway between the lowest rib and the iliac crest, after normal exhalation, using an anthropometric tape. Blood pressure was measured with the use of a validated semiautomatic oscillometer (Omron HEM-705CP, Netherlands) while the participant was in a seated position for 5 min.

Blood samples were collected after a 12-h overnight fast. Fasting plasma glucose, total cholesterol, high-density lipoprotein cholesterol (HDL-C), low-density lipoprotein cholesterol (LDL-C), and triglyceride concentrations were measured as previously described [107]. Leisure-time physical activity was assessed using the validated REGICOR questionnaire [108], including questions to collect information on the type of activity, frequency (number of days), duration, and total leisure-time physical activity-related energy expenditure was estimated. The dietary pattern was assessed by a 17-item score capturing adherence to a traditional Mediterranean diet [109]. We have published the detailed version of this questionnaire as well as its association with quality of life and cardiovascular risk factors [109]. Briefly, traditional Mediterranean diet is characterized by a high intake of vegetables, fruits, legumes, fish, as well as fat from vegetable sources, including nuts, and a low intake of red meat and desserts. In the 17-item screener, a suitable consumption of typical traditional Mediterranean foods adds one point and a low consumption of foods not characteristic of the Mediterranean diet also adds one point. A higher score means higher adherence to the Mediterranean diet. Supplemental Table S1 shows the 17 detailed items and the corresponding scores.

### 2.3. Serum Fatty Acid Determinations

Serum was obtained from fasting blood samples as previously detailed [55]. Serum fatty acids were measured by proton nuclear magnetic resonance (NMR) spectroscopy as previously reported in detail [110,111]. This high—throughput NMR metabolomics platform (Nightingale Health Ltd., Helsinki, Finland), has been applied and validated in numerous studies [112,113]. The measurements of PUFA with this NMR platform were only undertaken on participants recruited at the PREDIMED Plus-Valencia field center. Currently, there are no participants from other field centers with these data determined. In this study, we used all the available measurements of serum fatty acids provided by the platform. These included total fatty acids, monounsaturated fatty acids (MUFA), saturated fatty acids (SFA), total PUFA, omega-3 PUFA, and omega-6 PUFA. We also included LA and DHA for being the two specific fatty acids that that platform reported separately. No additional PUFA were reported in this platform. The results for total PUFA, omega-3, omega-6, and LA and DHA are very robust and have been validated in many publications [110,111,112,113,114,115]. Although we focused our genetic analyses on PUFA, for descriptive reasons, SFA, MUFA, and total fatty acids were also reported. Serum fatty acids were measured as follows: After measuring the lipoprotein data from native serum samples, their lipids were extracted and another NMR spectrum measured (see reference [116] for details). In these lipid extracts, each fatty acid gives rise to a characteristic NMR resonance, the area of which is associated with their concentration. After further scaling to account for slight experimental variation in lipid acquisitions in the extraction procedure [116], the different PUFA concentrations were quantified in mmol/L, representing the true concentration in serum. Finally, serum fatty acids were expressed as a percentage of total fatty acids. Although both measures (absolute and relative) can be used [117], we have analyzed PUFA and percentage because in previous works, fatty acids have been commonly used as percentage of total, and we want to compare our results with those obtained in other populations.

### 2.4. Genome-Wide Genotyping

We isolated genomic DNA from the buffy coat samples with the MagNaPure LC DNA Isolation kit (ROCHE Diagnostics). We measured the concentration and the quality of the isolated DNA by the PicoGreen instrument (Invitrogen Corporation, Carlsbad, CA, USA). After DNA-quality control, high-density genotyping was performed in samples passing this control using the Infinium OmniExpress-24 v1.2 BeadChip genotyping array (Illumina Inc., San Diego, CA, USA) at the University of Valencia, Valencia. Genotyping was undertaken according to the manufacturer’s protocol with appropriate quality standards. The Infinium OmniExpress v1.2 BeadChip captures 713,599 markers. Allele detection and genotype calling were performed in the Genome Studio genotyping module (Illumina, Inc., San Diego, CA, USA). Data cleaning was performed using standard analysis pipelines implemented in the Python programing language using the Numpy library modules combined with PLINK [118,119]. From the initial full set of SNPs in the array, those not mapped on autosomal chromosomes were filtered out. In addition, SNPs with a minor allele frequency (MAF) <0.01 or those that deviated from expected Hardy–Weinberg equilibrium (*p* < 1.0 × 10^−4^) were removed. A total of 621,723 SNPs that passed the quality filter remained for further analysis.

### 2.5. Statistical Analysis

Student t-tests and ANOVA tests were applied to compare crude means of continuous variables. Triglyceride and fatty acid concentrations (in mmol/L) were log-transformed for the statistical analyses. Fatty acids, expressed as % (total fatty acid of interest to total fatty acids), reached a normal distribution, and were not log transformed. Chi-square tests were used to compare proportions. Finally, we analyzed the omega-6/omega-3 ratio and we log transformed this variable to achieve normality for statistical testing. We analyzed the associations using both crude models and adjusted multivariate regression models (general lineal models) including potential confounders.

For the GWAS, at the genome-wide level, we tested the association between all the SNPs in the array (excluding those indicated above according to the quality control issues) and serum concentrations (expressed as %) of the different PUFA. We fitted separate models for omega-3 PUFA, DHA, omega-6 PUFA, LA, and total PUFA. Statistical models were sequentially adjusted by sex, age, diabetes, and other covariates including BMI, smoking, medications, physical activity, and adherence to Mediterranean diet (17-item score). Additional adjustment did not change the statistical level of significance. Therefore, for the sake of simplicity, and to compare our results with the previous GWAS carried out in different populations [79,80,81,82,83,84,85,86], that were mainly adjusted for sex and age, unadjusted models (Model 1) and multivariate models sequentially adjusted for sex, age, and diabetes (Model 2), were presented in tables and figures. Additional adjustments were specifically presented when indicated. For the study of genome-wide interactions with selected factors, hierarchical statistical general linear models were fitted. These models include the main variables and the corresponding interaction terms. Gene*sex and gene*diet genome-wide interactions were analyzed. For the study of the interactions between the SNPs and adherence to Mediterranean diet, we categorized baseline adherence to the Mediterranean diet (17-item screening) into two groups based on the sample median (8 points), defining 2 groups as either “Low” adherence to Mediterranean diet (0–8 points), or “High” adherence to Mediterranean diet (9–17 points). This cut-off point has been previously used by our group in another genome-wide interaction study [105].

For GWAS, genetic association analyses were performed using PLINK v1.9 [118,119]. Additive genetic models (unadjusted and multivariate adjusted when indicated) were fitted using a genotype dosage (0, 1, or 2 copies of the variant allele). Regression coefficients for the minor allele were estimated. Likewise, determination coefficients (r^2^) were also estimated. To test gene*sex and gene*diet interactions at the genome-wide level, we used the PLINK GxE tool and showed the statistical significance on the interaction terms, as well as the regression coefficients for each stratum. Stratified analyses of interest were additionally carried out when indicated. We used the conventional threshold of *p* < 5 × 10^−8^ for genome-wide statistical significance. Likewise, according to the conventional GWAS rules, SNPs with *p*-values below 1 × 10^−5^ were also considered suggestive of genome-wide significance. As these thresholds are very conservative for a small sample size, in selected analyses, SNPs with *p*-values below 5 × 10^−5^ are also shown in the tables so that they may be compared with other studies that have analyzed similar associations and may be included in meta-analyses. SNPs were rank-ordered according to the minimum *p*-value in the genetic models.

We used R Qqman R library [120] and Haploview (version 4.2) [121] to create Manhattan plots and to calculate linkage disequilibrium among the SNPs of interest. Quantile–quantile plot comparing the expected and observed *p*-values [121] was performed in the R-statistical environment.

In addition to the GWAS approach, to explore potential associated loci (candidates based on previous knowledge) which might fail to be identified at the GWAS level due to the strict threshold for the whole genome approach (*p* < 5 × 10^−8^ for genome-wide statistical significance), we carried out additional RWASs. In these analyses, we previously selected a candidate region of the genome containing the most relevant genes previously associated with PUFA and analyzed the associations with all the SNPs present in the selected region. Two candidate regions were selected [4,94]: (A) the region in chromosome 11 containing the *FASD1*/*FASD2*/*FASD3* cluster [94]; and (B) the region in chromosome 6 containing the *ELOVL2* and *ELOVL5* genes [4]. All the SNPs in the selected genes for each region, included in the Human OmniExpress array were analyzed for associations with the serum PUFA (omega-3, DHA, omega-6, LA, and total PUFA). The corresponding genotype data were extracted by a Python script, created by the authors. For selecting the available SNPs in these genes, we used another Python script, also created by the authors. For this procedure, we first used the batch query resource SNP Report with URL: https://www.ncbi.nlm.nih.gov/SNP/snp_ref.cgi?locusId, and provided the target gene-ID. The dbSNP-gene type file is used as input argument in the *.bat file for running the Python script. Finally, the Python script produces the corresponding PLINK-formatted couple *.ped and *.map files of genotypes for statistical testing. For the selected regions, we carried out unadjusted and multivariate adjusted analyses determining the serum PUFA, as previously detailed for the GWAS approach. Models adjusted for sex, age, and diabetes were presented. We also used PLINK v.1.9 [118,119] for these models. As the regional exploration is a replication analysis and our sample size is small, we considered a two-tailed *p*-value <0.05 as statistically significant for the RWAS associations.

## 3. Results

### 3.1. General Characteristics of the Population

Table 1 presents the demographic, lifestyle, and clinical characteristics of the study participants at baseline by sex. We analyzed 426 subjects, including 187 men and 239 women. These were all the subjects recruited and randomized in the Valencia field center having complete data of serum fatty acid determinations by NMR spectroscopy [110,111] and genome-wide genotyping available. These were individuals aged 65.1 ± 0.2 years, with metabolic syndrome. The average mean age of men and women was 64.0 ± 0.4 years and 66.0 ± 0.3 years (*p* < 0.001), respectively. BMI did not differ significantly in men and women. The women analyzed in this study were all post-menopausal women.

Table 1 also shows total fatty acid concentrations in serum determined in the NMR platform as well as MUFA, SFA, PUFA, omega-3, and omega-6. In addition to these total profiles, the platform also reported specific measures for LA and DHA. Fatty acid concentrations were expressed in mmol/L as well as in % (ratio of the corresponding fatty acid to the total fatty acids). We used PUFA expressed as % for further statistical analyses.

Total PUFA was 35.9 ± % of the total fatty acids and did not differ between men and women. Similar percentages of omega-3 PUFA (4.09% total; *p* = 0.182 for sex differences) and omega-6 PUFA (31.8% total; *p* = 0.723 for sex differences were detected in men and women). Likewise, the percentage of DHA and LA did not differ per sex. We also calculated the omega-6 to omega-3 ratio (no units for the ratio). This ratio was 7.98 ± 0.07 for the whole population and no statistically significant differences were detected between men and women (*p* = 0.254).

In these subjects with metabolic syndrome, consumption of antihypertensive drugs was high (78.4%). Consumption of lipid-lowering drugs (64.3%), consisting of mainly statins, was also high. No significant differences by sex were detected regarding for antihypertensive, lipid-lowering, or antidiabetic medications. In addition to the main medications presented in Table 1, Supplemental Table S2 shows additional medications taken (self-reported) in this population. Consumption of vitamins and minerals was reported for 12% of the populations, with significant differences (*p* < 0.001) between men (5.9%) and women (16.7%). There were no participants taking fish oil supplements. These supplements mainly consisted of calcium, folic acid, vitamin D, and some multivitamins (currently we do not have detailed information about their composition).

Mean adherence to the Mediterranean diet was 8.1 ± 0.1 point in the 17-item score. No statistically significant differences were detected between men and women (*p* = 0.145). We defined 2 groups of adherence to the Mediterranean diet as either “Low” adherence (0–8 points), or “High” adherence (9–17 points). This cut-off point has been previously used by our group for genome-wide-diet interaction studies [105].

We next focused on the genes and SNPs associated with the serum PUFA concentrations for all the 6 PUFA variables analyzed (omega-3 PUFA, DHA, omega-6 PUFA, LA, total PUFA, and the omega-6 to omega-3 ratio), using both a GWASs approach and a RWASs approach as detailed in Methods.

### 3.2. GWAS for Serum Omega-3 PUFA (%) in this Population

**Figure 1:**
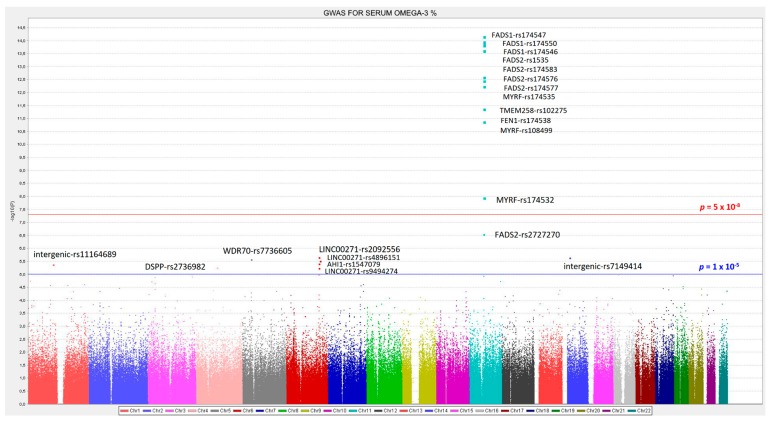
Manhattan plot for the genome-wide association study (GWAS) of serum omega-3 fatty acid (%) in the whole population. Associations were obtained from the genetic additive model and *p*-values were expressed as −log_10_ (*p*-value). The red line represents the threshold for GWAS statistical significance (−log_10_ (5 × 10^−8^)). The blue line represents the threshold for suggestive GWAS significance (−log_10_ (1 × 10^−5^)).

**Figure 2:**
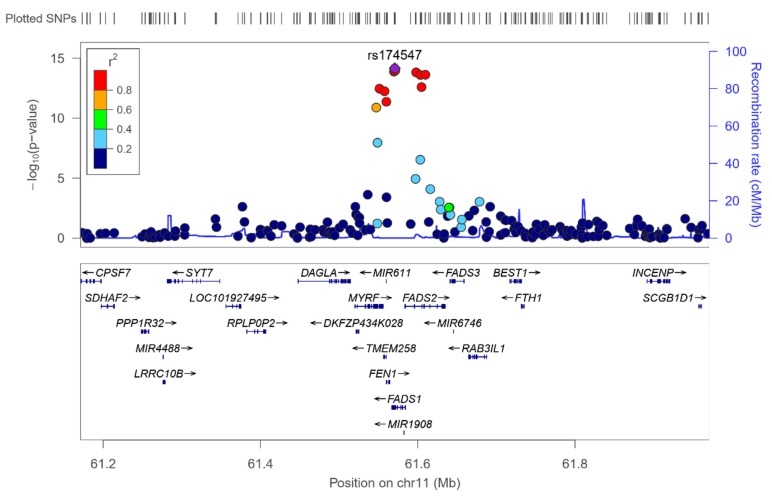
Regional plot for the top-ranked SNP rs174547 for serum omega-3 fatty acid, located in the *FADS1* gene, on chromosome 11. Results in the whole population.

We analyzed the associations at the genome-wide level in the whole population among all the tested SNPs (after quality control filtering) in the Illumina Human OmniExpress array and the 6 PUFA determined. For omega-3 PUFA, we obtained several very strong associations at the genome-wide statistical level of significance (*p* < 5 × 10^−8^), replicating previous findings in other populations. Figure 1 presents the corresponding Manhattan plot showing the *p*-value (−log10 *p*) of each SNP analyzed as well as indicating both thresholds for significance (the GWAS *p*-value of significance and the *p*-value suggestive of significance; *p* < 5 × 10^−5^). Supplemental Figure S2 shows the corresponding Q–Q plot. The top-ranked SNP was the *FADS1*-rs174547 with *p* = 7.02 × 10^−15^ in the unadjusted model (Figure 2 shows the regional plot for this SNP).

Table 2 presents more information of the top-ranked SNPs both in the crude (Model 1) and in the adjusted model for sex, age, and diabetes (Model 2). The variance explained by the hit SNP was high (13.4%) and this SNP remained as the top-ranked in the model adjusted for sex, age, and diabetes. Its MAF was also relatively high (0.298) and the effect (regression coefficient beta was −0.366 in the adjusted model). As we fitted an additive model, it means that each minor allele (C-allele) was associated with a decrease of the % of serum omega-3 PUFA. Other SNPs in the *FADS1* and *FADS2* genes also reached the GWAS level of significance even after adjustment for sex, age, and diabetes (*FADS1*-rs174550, *p* = 5.35 × 10^−14^; *FADS2*-rs1535, *p* = 5.85 × 10^−14^; *FADS1*-rs174546, *p* = 6.72 × 10^−14^; *FADS2*-rs174546, *p* = 9.75 × 10^−14^; *FADS2*- rs174576, *p* = 1.17 × 10^−13^; *FADS2*-rs174577, *p* = 1.12 × 10^−12^). Also, at the genome-wide level of significance, we detected other genes, close to the *FADS1*/*FADS2* cluster. These genes and SNPs being: *MYRF* (myelin regulatory factor)-rs174535, *p* = 1.49 × 10^−12^; *TMEM258* (transmembrane protein 258)-rs102275, *p* = 2.43 × 10^−12^; *FEN1* (flap structure-specific endonuclease 1)-rs174538, *p* = 1.96 × 10^−11^; *MYRF*-rs108499, *p* = 4.21 × 10^−11^; *MYRF*-rs174532, *p* = 1.20 × 10^−08^. Previous studies also have detected these SNPs as top-ranked in different populations (31, 37, 38). The SNPs near the *FADS1*/*FADS2* locus, were also highly associated with the *FADS1*/*FASD2* SNPs. 

**Figure 3:**
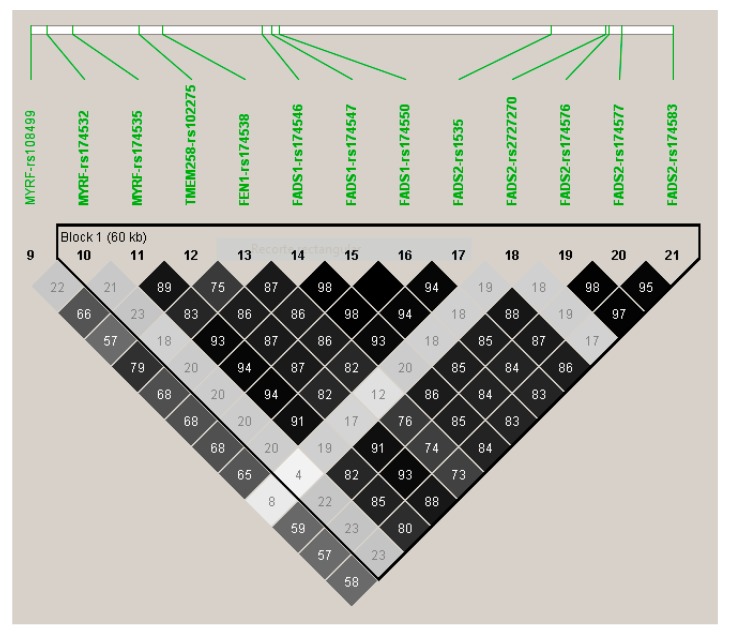
Linkage disequilibrium (r^2^) among the top-ranked SNPs (*p* < 5 × 10^−8^, in the model adjusted for sex, age, and diabetes) for the genome-wide association study on serum omega-3 fatty acid.

Figure 3 shows the LD (using r2) among the top-ranked SNPs. Apart from the *MYRF*-rs174532, and the *FADS2*-rs2727270, the other SNPs were highly correlated among them and with the lead SNP (*FADS1*-rs174547).

**Figure 4:**
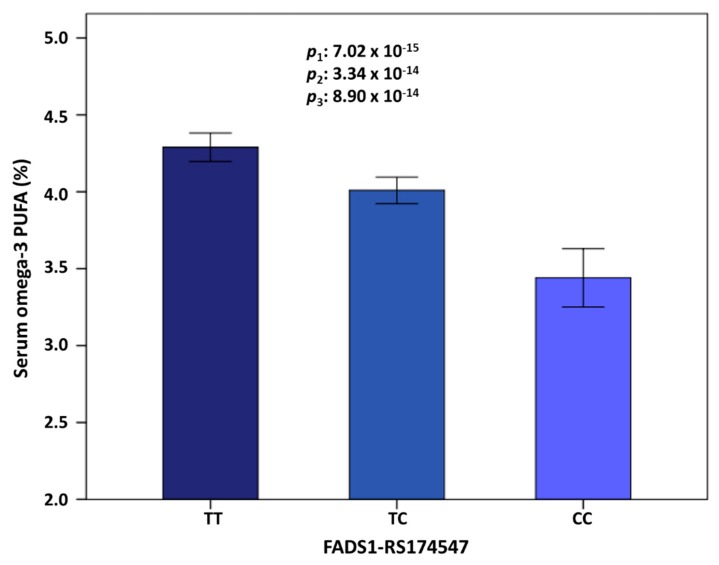
Serum omega-3 PUFA concentrations (%) depending on the *FADS1*-rs174547 genotype in the whole population. Genotype prevalence was: TT 49.3% (n = 212), TC 41.0% (n = 172), and CC 9.7% (n = 42). The minor allele (C) was associated with lower concentrations in all the adjusted models. 1: Unadjusted model; 2: Model adjusted for sex, age, and diabetes; 3: Model adjusted for sex, age, diabetes, body mass index, medications (antihypertensive, hypolipidemic, and antidiabetic drugs), smoking, physical activity, and adherence to Mediterranean diet. Values are means by genotype. Error bars: 2 × Standard Error.

Figure 4 shows the means of omega-3 PUFA (%) depending on the top-ranked *FADS1*-rs174547 polymorphism. In this Mediterranean population, the T allele was the major allele (this prevalence highly varies depending on the population studied). Minor allele carriers presented significantly lower serum omega-3 PUFA concentrations (TT: 4.29% ± 0.04; TC: 4.09% ± 0.05 and CC: 3.44% ± 0.1). This strong genetic association did not vary significantly even after multivariate association (*p* = 7.02 × 10^−15^ in the unadjusted model; *p* = 3.35 × 10^−14^ in the model adjusted for sex, age, and diabetes and *p* = 8.90 × 10^−14^, in the model adjusted for sex, age, diabetes, BMI, medications (including antihypertensive drugs, lipid lowering drugs, and insulin and oral antidiabetic medication), smoking, physical activity, and adherence to the Mediterranean diet. A previous GWAS in the Framingham study also showed a slight effect of the dietary adjustments in the SNP-PUFA associations [122]. Additional adjustment of the model containing all of these variables for more medications (including drug for pain and fever, anti-platelet drugs, tranquilizers-sedative-hypnotics, and vitamins/minerals), did not substantially change the statistical significance of the association between the *FADS1*-rs174547 polymorphism and serum omega-3 concentrations (*p* = 2.90 × 10^−13^).

In addition to the SNPs found at the genome-wide level of significance, we also detected several SNPs at the suggestive *p*-value of GWAS significance (*p* < 5 × 10^−5^). Among these genes, we would like to highlight the signal at the AHI1 (Jouberin) gene, located on chromosome 6, close to the MYB (proto-oncogene, transcription factor) gene and the HBS1L (like translational GTPase) gene, both of which have previously been reported in the Framingham study as new genes associated with PUFA [69]. However, no association signals at the GWAS level, or at the suggestive *p*-value of GWAS significance, were found for the *ELOVL* candidate genes on chromosome 6 for omega-3 PUFA in our Mediterranean population.

We next explored gene*sex interaction in determining serum omega-3 PUFA concentrations at the genome-wide level and only found a statistically significant interaction at the genome-wide level of statistical significance between the rs17097464-intergenic at chromosome 12 and sex (*p* = 1.13 × 10^−8^). The MAF for this SNP was 0.193. In addition to this hit, we found 7 other SNPs (rs11081343, rs1402508, rs8052428, rs4732314, rs897476, rs6499260, and rs11754354) reaching *p*-values < 1 × 10^−5^ for the interaction terms with sex. Future studies should investigate these loci in more detail.

**Figure 5:**
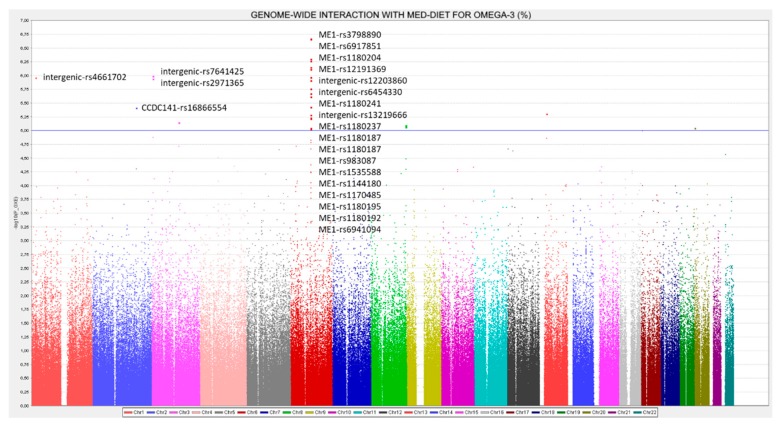
Manhattan plot for the genome-wide interaction with adherence to Mediterranean diet in determining serum omega-3 fatty acid (%) in the whole population. *p*-values for the gene*diet interaction terms were obtained in the additive model for the SNPs and diet expressed as dichotomous (low and high adherence to the Mediterranean diet) and expressed as −log_10_ (*p*-value). The blue line represents the threshold for suggestive GWAS significance (−log_10_ (1 × 10^−5^)).

In another exploratory analysis, we investigated gene* Mediterranean diet interactions in determining serum omega-3 PUFA concentrations. Two Mediterranean diet strata were considered (low and high adherence to Mediterranean diet). Figure 5 shows the Manhattan plot for these interactions.

**Figure 6:**
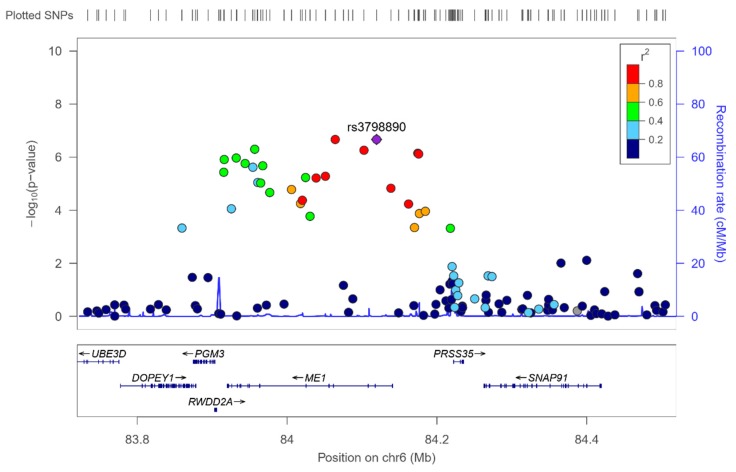
Regional plot for the top-ranked SNP rs3798890 (hit in the genome-wide interaction study with Mediterranean diet in determining serum omega-3 fatty acid), located in the *ME1* gene, on chromosome 6. Results for the whole population.

13 SNPs in the *ME1* (malic enzyme 1) gene significantly interacted (at *p* < 1 × 10^−5^) with adherence to Mediterranean diet with *p*-values ranging from 2.15 × 10^−7^ to 9.4 × 10^−6^ (see Supplemental Table S3 for the detailed SNPs, betas, MAF, genes, and *p*-values for the corresponding interaction terms). Supplemental Figure S3 shows the Q–Q plot for these interactions, indicating a good performance. The *ME1* gene is located on chromosome 6 and it is a plausible candidate for gene-diet interactions, taking into account that this gene is involved in fatty acids synthesis in the liver [123,124] and its modulation by the dietary fat content has been reported [124,125]. Figure 6 shows the regional plot for the top-ranked SNPs, *ME1*-rs3798890 (*p* = 2.15 × 10^−7^) in the genome-wide interaction analysis with Mediterranean diet. For this SNP as well as for the other SNPs top-ranked in the region, Supplemental Table S3 shows the interaction effect and the corresponding beta in the two strata of Mediterranean diet. Thus, when adherence to Mediterranean diet is low, the minor allele is associated with an increase in serum omega-3 PUFA concentrations, whereas when adherence to Mediterranean diet is high, the minor allele is associated with a decrease in serum omega-3 PUFA concentrations. This interaction effect might be similar to the previously reported positive selection for the *FADS* cluster [95], where depending on the diet, the prevalence of the allele resulting in the generation of higher PUFA concentrations, changed in the different populations.

### 3.3. GWAS for Serum DHA Concentrations (%) in this Population

**Figure 7:**
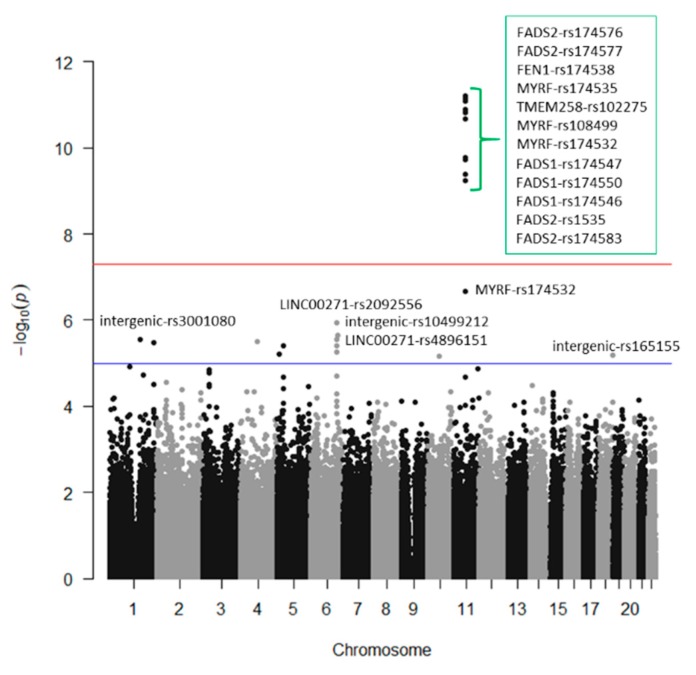
GWAS for serum docosahexaenoic fatty acid (%) in the whole population.

Figure 7 depicts the Manhattan plot for the GWAS on serum DHA concentrations (%) in this population and Table 3 shows the SNPs, MAFs, betas, determination coefficients, and *p*-values for the top-ranked SNPs in the models.

.

Supplemental Figure S4 presents the corresponding Q–Q plot. In general, a similar pattern of genes and polymorphisms were detected for DHA and total omega-3 and we also identified several associations at the genome-wide level of significance for DHA. Three SNPs in the *FADS1* gene (rs174547, rs174550, and rs174546) were the hits, having *p*-values of 1.28 × 10^−11^, 1.50 × 10^−11^, and 1.66 × 10^−11^). These SNPs were in very high LD. Likewise, 4 SNPs in the *FADS2* with *p*-values ranging from 2.87 × 10^−11^ to 3.47 × 10^−10^; 1SNP in *FEN1*-rs174538 (*p* = 3.47 × 10^−10^), 1 SNP in *TMEM258* (*p* = 8.79 × 10^−10^), and 2 SNPs in *MYRF*, with *p*-values of 3.89 × 10^−10^ and 1.22 × 10^−9^, were statistically significant at the genome-wide level. Despite their significance, the genetic effect for DHA was lower than that observed for total omega-3. Here, the top-ranked SNP (the *FADS1*), only explained the 10.6% of the variability, versus a 13.4%, explained by the same SNP for total omega-3. This indicates that here are other omega-3 fatty acids (not determined in this project) for which the genetic association effect should be higher.

In the exploratory analysis of the genome-wide interaction by sex in determining serum DHA concentrations, we found as top-ranked the same SNP that reached statistical significance at the GWAS level for the omega-3 PUFA. In this case, the interaction between the intergenic-rs17097464 on chromosome 12 and sex was 1.31 × 10^−7^. Likewise, consistent gene-sex interactions were found at *p* < 1 × 10^−5^ with several genes also detected at this level for the analysis on omega-3 PUFA. Among these genes, similar interaction results at (*p* < 1 × 10^−5^) have been found with *LOC105372018*-rs8091414; *NHEJ1* (non-homologous end joining factor 1)-rs1402508 and the intergenic-rs11081343.

Regarding the exploratory analysis of genome-wide interactions with adherence to Mediterranean diet, we also detected several interactions with the *ME1* gene in determining serum DHA concentrations. The top-ranked SNP for gene*Mediterranean diet interactions in the *ME1* was the rs1180204-*ME1* at *p* = 5.34 × 10^−7^.

### 3.4. GWAS for Serum Omega-6 Concentrations (%) in this Population

Supplemental Figure S5 displays the Manhattan plot for the GWAS associations in the whole population in determining serum omega-6 concentrations. Unlike that observed in GWAS for omega-3 PUFA, we did not detect any statistically significant association at the GWAS level for omega-6 PUFA. We only detected some associations at *p* < 5 × 10^−5^. Supplemental Figure S6 shows the corresponding Q–Q plot and Supplemental Table S4 [2], presents the top ranked SNPs for the omega-6 GWAS, both in the unadjusted and in the multivariate adjusted model. We can see that the genetic effect is lower for omega-6 PUFA compared to omega-3 PUFA in this population. The top-ranked SNP for omega-6 PUFA only accounted for 4.9% of variability in serum omega-6 concentrations, compared to 13.4% for omega-3 PUFA. Furthermore, top-ranked SNPs were not polymorphisms in the *FADS* genes, but in other genes. After adjustment for age, sex, and diabetes, the top-ranked SNP (rs4491485; *p* = 1.97 × 10^−6^) is located in the *ISG20* (interferon stimulated exonuclease gene 20) gene, followed by an intergenic SNP (rs2551402) in chromosome 18 and another SNP (rs949037; *p* = 4.84 × 10^−6^) located in the *BCL2* (BCL2 apoptosis regulator) gene, also in chromosome 18.

When we analyzed gene–sex interactions at the genome-wide level on omega-6 PUFA concentrations, we found no interaction at the GWAS significance level. However, the most significant interaction was obtained with the *DNTTIP2* (deoxynucleotidyltransferase terminal interacting protein 2) gene located at chromosome 1. The top ranked SNP was the *DNTTIP2*-rs3747965 at *p* = 1.07 × 10^−7^. Other SNPs in the *DNTTIP2* gene also achieved statistical significance at *p* < 5 × 10^−5^. Regarding, the exploratory analysis of gene*Mediterranean diet interactions on serum omega-6 concentrations, the top-ranked SNP was the *LOC105376941*-rs977905 at *p* = 1.17 × 10^−6^.

### 3.5. GWAS for Serum LA Concentrations (%) in this Population

Supplemental Figure S7 depicts the Manhattan plot for the GWAS on serum LA concentrations, and Supplemental Figure S8 shows the corresponding Q–Q plot. Two SNPs, the rs5994479 at the *C22orf42* and the *DAB1* (DAB adaptor protein 1) presented association at the GWAS level. This gene, involved in brain signaling in the endothelium, is an integrative signal essential for neuro-glia-vascular communication [126]. Supplemental Table S5 shows the descriptors and coefficients of these SNPs. In the model adjusted for sex, age, and diabetes, the significance achieved by each of them was 5.25 × 10^−10^ for rs5994479 and 5.69 × 10^−10^ for *DAB1*-rs11207162. Although the variability explained by the top-ranked SNP is greater for LA (9.5%) than for total omega-6, it is still lower than for DHA. It is also striking that none of the candidate genes in the *FADS* or *ELOVL* genes are among the top-ranked.

In the genome-wide interaction study to analyze the interactions with sex, we obtained several significant associations at the *p* < 1 × 10^−5^ with SNPs in the *DNTTIP2* gene. This is highly consistent with that observed for total omega-6. Likewise, the top ranked gene–sex interaction for serum LA concentrations was observed with the gene *DNTTIP2*-rs3747965 at 1.04 × 10^−7^ level. For the genome-wide interactions with adherence to Mediterranean diet on serum LA, the top-ranked SNP was *LOC105374410*-rs1906292 at *p* = 8.88 × 10^−7^. This SNP was also the hit for omega-6 PUFA.

### 3.6. GWAS for Serum Total PUFA Concentrations (%) in this Population

In the GWAS for total PUFA, we only detected 4 statistically significant SNPs at *p* < 5 × 10^−5^ in the sex-, age-, and diabetes-adjusted model. None of them reached the level of GWAS significance and the *p*-values were higher than 3.12 × 10^−6^. This may reflect the greater heterogeneity of this variable. Among the most significant SNPs are those already detected as top-ranked for omega-6 or LA, as omega-6 are the most abundant PUFA. These SNPs were: Intergenic-rs7047109 (3.12 × 10^−6^), *DNAH11* (dynein heavy chain 11, axonemal)-rs10256021 (*p* = 4.8 × 10^−6^); *ISG20*-rs4491485 (*p* = 7.18 × 10^−6^) and intergenic-rs252181 (*p* = 9.69 × 10^−6^).

**Figure 8:**
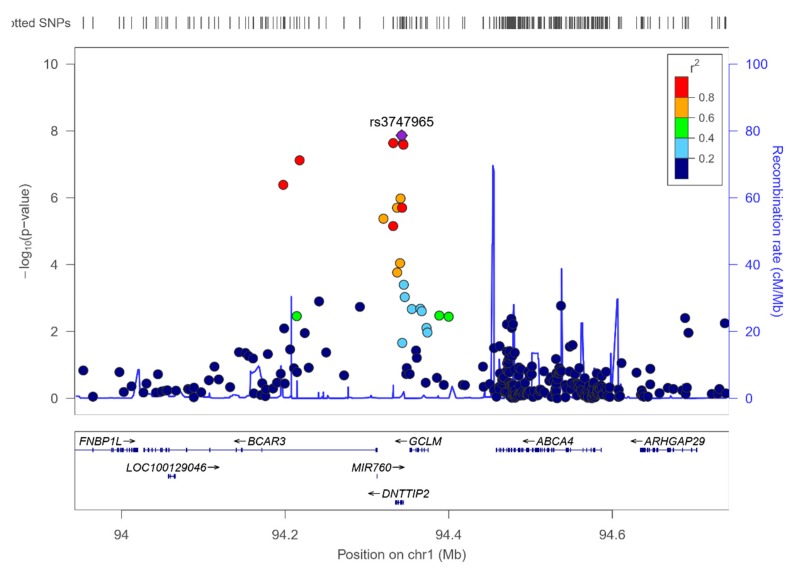
Regional plot for the top-ranked SNP rs3747965 (hit in the genome-wide interaction study with sex in determining serum polyunsaturated fatty acids), located in the *DNTTIP2* gene, on chromosome 1. Results for the whole population.

Interestingly, in the study of genome-wide interaction with sex, the top-ranked SNP determining the concentrations of total PUFA was the *DNTTIP2*-rs-3747965, already obtained in the previous analyses for omega-6 and for LA, but this time, reaching the level of statistical significance of GWAS (*p* = 1.36 × 10^−8^). Other SNPs in the *DNTTIP2* gene and nearby, also reached the genome-level of significance. Supplemental Figure S9 shows the Manhattan plot for the gene*sex interaction terms in determining serum total PUFA, and Figure 8, shows the regional plot for the top-ranked SNP *DNTTIP2*-rs-3747965.

According to this interaction, the minor allele of this SNP (MAF: 0.345) was associated with reduced PUFA in females (beta: −0.634) and with increased PUFA in males (beta: 1.158).

### 3.7. GWAS for the Omega-6 to Omega 3 Ratio

We also explored at the GWAS level the SNPs associated with the omega-6 to omega-3 ratio in this population. Supplemental Table S6 shows the top-ranked SNPs associated with this ratio (log transformed). Both in the unadjusted and in the multivariate adjusted models (for sex, age, and diabetes), several SNPs in the chromosome 11 (*FADS1/2* cluster) showed statistically significant associations with the omega-6/omega-3 ratio at the GWAS level. The top-ranked SNP was the *FADS1*-rs174547 T > C in both models. Specifically, in the multivariate adjusted model, the minor allele of this SNP was associated with a higher omega-6/omega-3 ratio (B = 0.041; *p* = 2.19 × 10^−14^).

Supplemental Figure S10 shows the means of the serum omega-6/omega-3 ratio (log transformed) depending on the top-ranked *FADS1*-rs174547 polymorphism. Homozygous subjects for the minor allele (CC) presented significantly higher ratio. This strong genetic association changed little even after multivariate adjustment (*p* = 3.90 × 10^−15^ in the unadjusted additive model; *p* = 2.19 × 10^−14^ in the model adjusted for sex, age, and diabetes and *p* = 9.81 × 10^−14^, in the model adjusted for sex, age, diabetes, BMI, medications (including antihypertensive drugs, lipid lowering drugs, and insulin and oral antidiabetic medication), smoking, physical activity, and adherence to the Mediterranean diet. Additional adjustment of the model containing all of these variables for more medications (including drug for pain and fever, anti-platelet drugs, tranquilizers-sedative-hypnotics, and vitamins/minerals), did not substantially change the statistical significance of the association (*p* = 1.71 × 10^−13^). Furthermore, we analyzed the interaction between the adherence to the Mediterranean diet and the SNPs at the genome-wide level in determining the serum omega-6/omega-3 ratio (Supplemental Figure S11). We obtained similar results for this interaction than those described for the serum omega-3 PUFA concentrations. Thus, the top-ranked gene interacting with adherence to the Mediterranean diet was the *ME1* gene. More studies are needed to replicate and to characterize this interaction.

### 3.8. RWASs for Serum PUFA Concentrations

**Figure 9:**
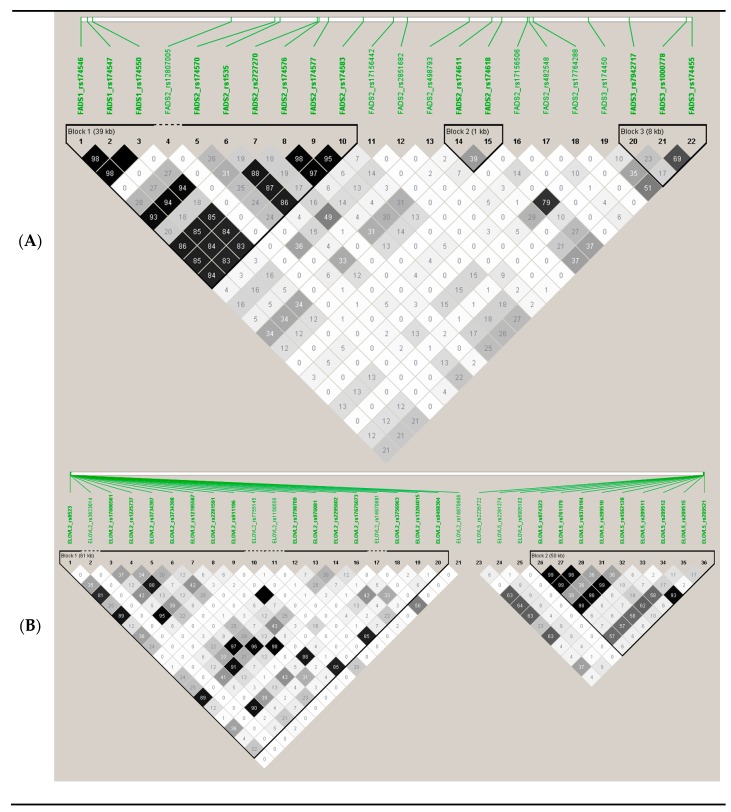
Genes, SNPs, and linkage disequilibrium (r2) among the SNPs selected for the regional-wide association studies (RWASs), based on previous knowledge of the relevance of this region for the associations between its SNPs and PUFA concentrations: (**A**) region containing all the SNPs in/near the cluster *FADS1*/*FADS2*/*FADS3* (chromosome 11) in the Illumina HumanOmniexpress array; (**B**) region containing all the SNPs in/near the cluster *ELOV2*-*ELOV5* (chromosome 6) in the Illumina HumanOmniexpress array.

To explore potential associated loci which might fail to be identified due to the strict threshold for GWAS associations, we carried out a RWAS investigating 2 regions previously reported to be genetic determinants of the SNPs analyzed. Figure 9 shows the SNPs analyzed in region A (*FADS* cluster) and in region B (*ELOVL2*/*ELOVL5*).

Supplemental Table S7 presents the SNPs and the associations obtained in the RWAS analysis for serum omega-3 at the *FADS1*/*FADS2*/*FADS3* region. We obtained statistically significant associations at *p* < 0.05 with 15 SNPs (models adjusted for sex, age, and diabetes), including SNPs detected at the GWAS level and other SNPs detected at the nominal level of significance. In addition to the *FADS1* and *FADS2* SNPs, we detected a significant association (*p* = 0.012) with the *FADS3*-rs174450 SNP and with the *FADS3*-rs174455 (*p* = 0.027). Supplemental Table S8 shows the corresponding RWAS associations with DHA. Similar results were obtained, and 12 SNPs were detected at *p* < 0.05. However, no statistically significant association even at this level with *FADS3* SNPs was observed.

For omega-6 PUFA, the *FADS1*/*FADS2*/*FADS3* region was poorly associated with their serum levels in this population even after considering the nominal *p*-value. Supplemental Table S9 shows these associations. Only 2 SNPs *FADS2*-rs174618 and *FADS2*-rs174611, reached the significance at *p* = 0.037 and *p* = 0.047, respectively. However, for LA concentrations, 11 SNPs in the analyzed *FADS1*/*FADS2*/*FADS3* region, reached statistical significance at *p* < 0.05 (Table 4). The most significant SNP was the *FADS2*-rs174611 (*p* = 0.0005).

For total PUFA, the RWAS at the *FADS1*/*FADS2*/*FADS3* region revealed 6 SNPs (rs2727270, rs174547, rs174550, rs174546, rs1535, and rs174570) statistically associated with serum levels at *p* < 0.05.

For region B, consisting of SNPs in *ELOVL2* and *ELOVL5*, we did not detect any statistically significant association with serum omega-3 PUFA (Supplemental Table S10). Similar results were obtained for DHA (no statistically significant associations). However, for omega-6 PUFA (Supplemental Table S11) and LA (Supplemental Table S12), some statistically significant associations involving *ELOVL2* SNPs were found. For total PUFA concentrations, no statistically significant associations with region B SNPs were found.

## 4. Discussion

In this first GWAS carried out in a Mediterranean population composed of subjects with metabolic syndrome in Spain, we confirmed the large contribution of the *FASD1* gene cluster (chromosome 11) on total serum omega-3 PUFA [94]. Despite the limited sample size, our findings show robust statistically significant associations with *p* < 5 × 10^−8^ for several SNPs in/near the *FASD1* and *FASD2* genes. The top-ranked SNP was the *FADS1*-rs174547 T > C. This SNP was strongly associated with serum omega-3 concentrations even after additional adjustment for covariates (*p* = 3.34 × 10^−14^ in the model adjusted for sex, age, and diabetes). Further adjustments for medications, diet, and other lifestyle variables did not substantially change the statistical significance of the association. Likewise, some previous GWAS [81,83,96] only adjusted the main statistical models for age and sex (and recruitment site in multicenter studies) and further evaluated the effect of additional adjustment for other covariates (dietary intake, physical activity, and BMI). After these additional adjustments, the PUFA-SNP associations changed little, in agreement with our results testing sequential adjustments. In our population, the minor allele of the *FADS1*-rs174547 T > C top-ranked SNP (C allele) was associated with decreased serum omega-3 PUFA concentrations. This is consistent with functional studies showing a functional association between the *FADS1*-rs174547 and delta-5 desaturase activity [127]. Moreover, a recent study examining the effects of aging and FADS polymorphisms on long chain PUFA biosynthetic capacity via direct quantification [128] has reported for the first time that aging especially decreases the long chain PUFA biosynthetic capacity regulated by the *FADS1*-rs174547 SNP in *FADS1* C allele carriers [128]. The fact of studying an elderly population (subjects aged 55 to 75 years) is another factor that may have contributed to the differences detected between our study and others previously carried out [79,80,81,82,83,84,85,86,93,98].

In our GWAS, in addition to the association with serum omega-3 PUFA, we have studied the association with serum concentrations of DHA, an omega-3 fatty acid derived primarily from fish. For DHA we have also obtained statistically significant associations at the GWAS level with several SNPs in the genes *FADS1* and *FADS2* in agreement with the previous GWAS analyzing DHA concentrations, with *FADS1*-rs174547 SNP also being the top-ranked.

However, we did not detect statistically significant associations between the *FADS1*/*FADS2* cluster SNPs at the GWAS level with total serum omega-6 PUFA or LA (omega-6). This result differs from other GWAS studies [79,80,81,82,83,84,85,86]. The lack of association at the GWAS level of the *FADS1*/*FADS2* SNPs with serum omega-6 may be due to several factors. One of them may be related to the age of our population. We have studied older subjects (mean age 65 years old), and it has been reported that aging decreases biosynthetic activity regulated by the *FADS1* polymorphisms [128], so potentially decreasing the magnitude of the genetic associations at this locus. Along these lines, another factor influencing the activity of the *FADS1*/*FADS2*/*FADS3* desaturases is the ethnic background [95]. Our Spanish Mediterranean population may differ from the majority of the other populations included in the previously published GWAS [80,81,82,83,84,85,86]. Moreover, several environmental factors may contribute to the differences. Among them, the dietary pattern may play a relevant role. The existing GWAS have been mainly carried out in American populations [81,83,96] as well as in Asian populations [84,86] having different diets. The Spanish Mediterranean population has been characterized over many years as one that follows the so-called traditional Mediterranean diet which is rich in vegetables, fruit, fish, olive oil and nuts, and low in red meats, butter, pastries and processed products [99]. Nowadays, the younger population is turning away from the Mediterranean diet, but a fairly high adherence, on average, to the traditional Mediterranean diet can still be detected in the elderly, although variability in adherence also exists within this group [99,101]. Among the populations included in the previous GWAS for circulating PUFA, the one that most resembles our population is the INCHIANTI study [79], focused on an Italian Mediterranean population. In the INCHIANTI study, a GWAS was carried out on plasma levels of six omega-3 and omega-6 PUFA in 1075 participants. In this study, the strongest evidence for global associations was found at the *FADS1*/*FADS2*/*FADS3* cluster. This is similar to our results. However, in the INCHIANTI study, the associations were mainly observed for some omega-6 PUFA [79]. Thus, the SNP with the most significant association was rs174537 near *FADS1*, in determining plasma arachidonic acid (AA) concentrations (*p* = 5.95 × 10^−46^). In our study, we did not analyze the association with AA, because the NMR platform used for measuring serum fatty acids [110,111,112,113,114,115,116] did not provide the separate concentration of AA. Therefore, we cannot compare the results and this is a limitation of our study in comparison with other studies providing a more comprehensive profile of fatty acids determined in other platforms (80,81,83,84,86). However, in INCHIANTI, total omega-3 or total omega-6 PUFA were not analyzed. In the INCHIANTI paper [79], the authors compared their results with those obtained in the GOLDN (Genetics of Lipid Lowering Drugs and Diet Network) study including American subjects (n = 1076) recruited in Minneapolis, MN and Salt Lake City, UT. For some associations, replications were found, but other associations were population specific. This observation was in agreement with our initial hypothesis regarding the interest in analyzing specific populations to better understand the associations. Again, diet could be an important driver, taking into account the differences in the diet consumed when comparing INCHIANTY and the GOLDN populations. Additionally, the authors, pointed out that the tissue selected for PUFA determination may be another cofounder when results are compared. In GOLDN, PUFA were measured in erythrocyte membranes and in INCHIANTI, PUFA were measured in plasma [79]. It has been reported that erythrocytes and plasma may reflect two slightly different pools of fatty acids [129]. In general, it is accepted that plasma fatty acids reflect a short term fatty acid intake whereas erythrocyte levels reflect a more long term intake. This limitation is also present in the studies meta-analyzing populations with different fatty acid determinations and may have an influence when comparing results [130]. Moreover, an additional limitation, even in studies analyzing plasma fatty acids, is the type of measurement. Thus, Wu et al. [83] in the meta-analysis that they carried out in the CHARGE consortium outlined this potential limitation, specifically indicating that the INCHIANTI cohort measured total plasma fatty acids while the other 4 cohorts (ARIC, CARDIA, CHS, and MESA), measured plasma phospholipid fatty acids, and whether the scale of these different measurements is completely the same is unclear.

Another factor that may be related to some differences among studies when comparing GWASs is the medication use in the different populations. In previous GWAS [79,80,81,82,83,84,85,86], no adjustment for medications has been reported. In our study, we fitted statistical models sequentially adjusted for sex, age, and diabetes in order to compare our results with the previously published studies having similar adjustment. Additionally, we adjusted our main results for medications and this adjustment changed little the associations. However, an influence of lipid-lowering drugs (mainly statins) on PUFA levels has been reported in other studies [131,132]. Therefore, additional analysis with a large sample size are needed to better understand the role (and potential gene*statins interactions) of the medications in determining PUFA concentrations.

In addition to these limitations, sample size is another limitation for our study. Considering the strict threshold for statistically significant results at the GWAs level, some potential associations with relevant loci, might fail to be identified. Therefore, we conducted a RWAS, focused on previously identified regions considered as relevant for the genetic associations with PUFA [4,94]. The selected regions were the *FADS1*/*FADS2*/*FADS3* [94] cluster and the *ELOVL2*/*ELOVL5* [4] region. After testing the SNPs in the candidate genes in these regions, we detected statistically significant associations with LA concentrations that were not detected as significant signals at the GWAS level, supporting the associations between some *FADS1*/*FADS2*/*FADS3* SNPs and LA concentrations also in this population, but of a lower magnitude than the associations found for DHA or total omega-3 PUFA. Regarding the *ELOVL* region [4], although other studies have reported associations with several SNPs in these genes [79,81,82,83,84], in this Mediterranean population, the specific associations have been smaller, and even at the RWAS level, no significant associations have been detected for omega-3 PUFA. Only a few *ELOVL2* SNPs presented significant associations at *p* < 0.05 with total serum omega-6 and LA.

These results emphasize the need to carry out specific studies to know which genetic variants are most relevant in each population, because the results of the most significant associations in a population or in a meta-analysis are not always those that are actually observed in a different population [89]. In the case of the genetics of serum PUFA, it is known that due to the relevance that these fatty acids have for the functioning of the organism [94], the prevalence of the different genetic variants in the *FADS1*/*FASD2* genes has been adapting to changes in the diet, with the aim of achieving the highest concentrations of long chain PUFA [96]. Several studies have analyzed the details of this so-called positive selection [95,96,97,98], and currently, the prevalence of genetic variants and the overall effect of enzymes may be different depending on the population analyzed [95,96,97,98].

Some GWAS have considered dietary intake in adjustments carried out in the statistical analysis, indicating a modest influence [122]. However, to better understand dietary modulation, a gene*diet interaction analysis should be carried out. Although some GWAS and various candidate gene studies have examined the interaction between several dietary components (food or nutrients) and the genetic variants in determining PUFA concentrations [1,60,83,129,130,131,132,133,134,135], the interaction with a whole dietary pattern such as the Mediterranean diet pattern [99], has not been comprehensively investigated. In an exploratory study, taking into account our limited sample size, we explored gene* Mediterranean diet interactions at the genome-wide level in determining serum PUFA. Two categories (low and high adherence to Mediterranean diet) were analyzed using the same approach that we previously carried out in another genome-wide-interaction study with Mediterranean diet in the same population in determining plasma bilirubin concentrations [105]. The most relevant gene*Mediterranean diet interaction was found for the *ME1* gene in determining serum omega-3 concentrations. Several SNPs in this gene, having different MAF, reached statistical significance for the interaction term at *p* < 5 × 10^−5^. According to this interaction, the association between the *ME1* genotypes and serum omega-3 depends on the adherence to the Mediterranean diet pattern. Moreover, when we further analyzed the Mediterranean diet interaction at the GWAS level in determining the serum omega-6/omega-3 ratio, we also obtained the *ME1* as the top-ranked gene, replicating the results for omega-3 PUFA. This is the first time that this interaction is reported in humans and subsequent studies are needed to replicate and characterize the modulation. However, existing literature initially supports a potential mechanism behind this interaction. The *ME1* gene encodes a cytosolic, NADP-dependent enzyme that produces NADPH for fatty acid biosynthesis [124]. Likewise, the *ME1* gene has been implicated in PUFA biosynthesis [136,137,138]. Furthermore, in animal models, the *ME1* gene expression is strongly regulated by the dietary fat content [125,139]. Fat content of the low or high adherence group (high adherence characterized by a high fish intake, nuts, olive oil, and a low intake of red meat and other products rich in SFA) may modulate the effects of the *ME1* polymorphisms on serum omega-3 PUFA (as well as on the omega-6/omega-3 ratio), contributing to explain the statistical signal. Our findings are exploratory and additional replication studies are needed to confirm this interaction.

Likewise, genetic modulation by sex may be another relevant factor explaining some differences in the effect of the genetics of PUFA. Some PUFA are specifically required for reproduction [104]. It has been reported that PUFA concentrations are higher in females than in males during pregnancy and there are some studies showing sex-specific effects for some PUFA in determining several outcomes [68,69,70,71,72,73,74,140,141]. Although this is still an emerging field, the promotion of research investigating sex-specific differences [142] could provide more information regarding sex-specific effects on gene*sex interactions in determining PUFA levels for a more personalized nutrition. Our genome-wide gene*sex interaction study, has revealed a statistically significant gene*sex interaction involving the *DNTTIP2* gene (rs3747965) in determining serum total PUFA (mainly omega-6 PUFA), at the genome-wide level of significance (*p* < 5 × 10^−8^). Again, this is the first time that this statistical interaction has been reported, and more studies are needed to replicate and characterize this interaction. The *DNTTIP2* gene has the alternative name of “estrogen receptor-binding protein gene”, as well as the short name: “TdIF2” (terminal deoxynucleotidyltransferase interacting factor 2). The TdIF2/estrogen receptor α-binding protein (ERBP) is a multifunctional nucleolar protein. It promotes ribosomal RNA transcription by still partially unknown mechanisms [143]. Thus, there is a potential link between the SNP in this gene and estrogens [143], explaining the potential sex-specific interaction on PUFA. The literature examining the *DNTTIP2* is scarce, but in a recent GWAS, the *DNTTIP2* gene has been linked to ischemic stroke [144], an outcome potentially linked to the PUFA modulations. As stated before, this finding is only exploratory, and more research has to be done for replication and characterization. Overall, our novel findings provide a roadmap for further studies analyzing gene*sex and gene*diet interactions on PUFA concentrations in different populations.

## 5. Conclusions

In conclusion, our results allowed us to define the genetic variants most associated with serum PUFA concentrations in a Mediterranean population with metabolic syndrome and to confirm the strong association between the *FADS1*/*FADS2* locus and omega-3 PUFA (specifically DHA) in this population. The associations of this locus with omega-6 were much weaker, but also present at the RWAS level. Likewise, we have observed a strong genetic influence of the *FADS1*/*FADS2* cluster in determining the omega-6/omega-3 ratio, at the GWAS level, in this population. We have also detected some new interactions with sex (on omega-6 PUFA) and adherence to the Mediterranean diet (on omega-3 PUFA), which will have to be replicated and functionally characterized in other studies. This underlines the need for these variables to be studied in all specific populations, so as to better understand the complex metabolism of PUFA and their repercussions on health.

## Figures and Tables

**Table 1:** Demographic, clinical, lifestyle, and biochemical characteristics of the study participants at baseline according to sex.

Variables	Total (n = 426)	Men (n = 187)	Women (n = 239)	*p*
Age (years)	65.1 ± 0.2	64.0 ± 0.4	66.0 ± 0.3	<0.001
Weight (Kg)	84.2 ± 0.7	92.5 ± 1.0	77.7 ± 0.6	<0.001
BMI (Kg/m^2^)	32.3 ± 0.2	32.2 ± 0.3	32.3 ± 0.2	0.836
Waist circumference (cm)	105.8 ± 0.5	110.9 ± 0.6	101.8 ± 0.6	<0.001
SBP (mm Hg)	141.9 ± 0.9	144.1 ± 1.4	140.1 ± 1.2	0.029
DBP (mm Hg)	81.0 ± 0.5	82.7 ± 0.7	79.6 ± 0.6	0.001
Total cholesterol (mg/dL)	196.4 ± 1.8	188.3 ± 2.9	202.7 ± 2.3	<0.001
LDL-C (mg/dL)	124.9 ± 1.5	121.9 ± 2.4	127.2 ± 1.9	0.078
HDL-C (mg/dL)	51.6 ± 0.6	47.3 ± 0.8	55.0 ± 0.7	<0.001
Triglycerides (mg/dL)	140.7 ± 2.9	137.6 ± 3.9	143.1 ± 4.1	0.334
Fasting glucose (mg/dL)	112.4 ± 1.3	113.5 ± 2.1	111.5 ± 1.6	0.441
Total fatty acids (mmol/L)	11.09 ± 0.10	10.63 ± 0.14	14.46 ± 0.14	<0.001
SFA (mmol/L)	4.02 ± 0.04	3.85 ± 0.05	4.16 ± 0.05	<0.001
MUFA (mmol/L)	3.09 ± 0.03	2.97 ± 0.04	3.20 ± 0.05	<0.001
PUFA (mmol/L)	3.97 ± 0.04	3.81 ± 0.05	4.10 ± 0.05	<0.001
Omega-3 (mmol/L)	0.46 ± 0.01	0.43 ± 0.01	0.48 ± 0.01	<0.001
Omega-6 (mmol/L)	3.52 ± 0.03	3.38 ± 0.04	3.63 ± 0.04	<0.001
DHA (mmol/L)	0.146 ± 0.002	0.140 ± 0.003	0.150 ± 0.003	0.013
LA (mmol/L)	2.88 ± 0.03	2.78 ± 0.04	2.97 ± 0.04	0.001
SFA (%) ^1^	36.27 ± 0.06	36.25 ± 0.09	36.29 ± 0.08	0.722
MUFA (%) ^2^	27.82 ± 0.09	27.85 ± 0.13	27.79 ± 0.13	0.754
PUFA (%) ^3^	35.90 ± 0.11	35.89 ± 0.16	35.91 ± 0.15	0.950
Omega-3 (%) ^4^	4.09 ± 0.03	4.04 ± 0.05	4.13 ± 0.05	0.182
Omega-6 (%) ^5^	31.80 ± 0.10	31.85 ± 0.15	31.78 ± 0.14	0.723
DHA (%) ^6^	1.30 ± 0.01	1.31 ± 0.02	1.30 ± 0.02	0.710
LA (%) ^7^	26.04 ± 0.11	26.18 ± 0.18	25.90 ± 0.15	0.272
Omega-6/Omega-3 ratio	7.98 ± 0.07	8.07 ± 0.10	7.91 ± 0.09	0.254
Type 2 diabetes: n, %	160 (37.6)	70 (37.4)	90 (37.7)	0.962
Current smokers: n, %	46 (10.8)	28 (15.0)	18 (7.5)	<0.001
Antihypertensive drugs	334 (78.4)	148 (79.1)	186 (77.8)	0.742
Hypolipidemic drugs	274 (64.3)	121 (64.7)	153 (64.0)	0.883
Metformin treatment	128 (30.0)	56 (29.9)	72 (30.1)	0.968
Insulin treatment	19 (4.5)	11 (5.9)	8 (3.3)	0.208
Physical Activity (MET.min/wk)	1724 ± 76	1950 ± 132	1547 ± 85	0.008
Adherence to MedDiet (P17) ^8^	8.1 ± 0.1	7.8 ± 0.2	8.2 ± 0.2	0.145

Values are number and %. BMI indicates body mass index; MedDiet, Mediterranean diet; *p*: *p*-value for the comparisons (means or %) between men and women; PUFA: Polyunsaturated fatty acid; MUFA: Monounsaturated fatty acid; SFA: Saturated fatty acid; LA: Linoleic fatty acid; DHA: Docosahexaenoic fatty acid; MET: Metabolic equivalent. ^1^: Ratio of saturated fatty acid to total fatty acid. ^2^: Ratio of monounsaturated fatty acid to total fatty acid. ^3^: Ratio of polyunsaturated fatty acid to total fatty acid. ^4^: Ratio of omega-3 fatty acid to total fatty acid. ^5^: Ratio of omega-6 fatty acid to total fatty acid. ^6^: Ratio of docosahexaenoic fatty acid to total fatty acid. ^7^: Ratio of linoleic fatty acid to total fatty acid. ^8^: Quantitative 17-item questionnaire for adherence to Mediterranean diet.

**Table 2:** Top-ranked single nucleotide polymorphisms (SNPs) in the genome-wide association study (GWAS) for serum omega-3 fatty acid concentrations (%) in the whole population.

Unadjusted ^1^										Adjusted ^2^					
Chr	SNP	Gene	Position ^3^	Allele	MAF	Beta ^1^	SE ^1^	R^2^ ^(1)^	*p* ^1^	Chr	SNP	Gene	MAF	Beta ^2^	*p* ^2^
11	rs174547	*FADS1*	61570783	C	0.298	−0.372	0.046	0.134	7.02 × 10^−15^	11	rs174547	*FADS1*	0.298	−0.366	3.34 × 10^−14^
11	rs174550	*FADS1*	61571478	C	0.298	−0.369	0.046	0.132	1.10 × 10^−14^	11	rs174550	*FADS1*	0.298	−0.364	5.35 × 10^−14^
11	rs174546	*FADS1*	61569830	T	0.298	−0.368	0.046	0.131	1.34 × 10^−14^	11	rs1535	*FADS2*	0.322	−0.356	5.85 × 10^−14^
11	rs1535	*FADS2*	61597972	G	0.322	−0.361	0.045	0.131	1.51 × 10^−14^	11	rs174546	*FADS1*	0.298	−0.362	6.72 × 10^−14^
11	rs174583	*FADS2*	61609750	T	0.369	−0.352	0.044	0.129	2.38 × 10^−14^	11	rs174583	*FADS2*	0.369	−0.346	9.75 × 10^−14^
11	rs174576	*FADS2*	61603510	A	0.363	−0.352	0.045	0.129	2.49 × 10^−14^	11	rs174576	*FADS2*	0.363	−0.346	1.17 × 10^−13^
11	rs174577	*FADS2*	61604814	A	0.392	−0.339	0.045	0.119	2.56 × 10^−13^	11	rs174577	*FADS2*	0.392	−0.332	1.12 × 10^−12^
11	rs174535	*MYRF*	61551356	C	0.340	−0.344	0.046	0.117	3.56 × 10^−13^	11	rs174535	*MYRF*	0.340	−0.339	1.49 × 10^−12^
11	rs102275	*TMEM258*	61557803	C	0.493	−0.338	0.045	0.116	5.79 × 10^−13^	11	rs102275	*TMEM258*	0.493	−0.332	2.43 × 10^−12^
11	rs174538	*FEN1*	61560081	A	0.282	−0.346	0.048	0.107	4.25 × 10^−12^	11	rs174538	*FEN1*	0.282	−0.339	1.96 × 10^−11^
11	rs108499	*MYRF*	61547237	T	0.288	−0.332	0.048	0.103	1.31 × 10^−11^	11	rs108499	*MYRF*	0.288	−0.327	4.21 × 10^−11^
11	rs174532	*MYRF*	61548874	A	0.100	0.267	0.046	0.074	1.12 × 10^−08^	11	rs174532	*MYRF*	0.100	0.267	1.20 × 10^−08^
11	rs2727270	*FADS2*	61603237	T	0.163	−0.402	0.077	0.060	2.88 × 10^−07^	11	rs2727270	*FADS2*	0.163	−0.391	8.01 × 10^−07^
6	rs2092556	*LINC00271*	135899344	C	0.188	0.409	0.085	0.052	2.23 × 10^−06^	6	rs2092556	*LINC00271*	0.188	0.413	1.85 × 10^−06^
14	rs7149414	intergenic	31237170	A	0.133	0.381	0.080	0.052	2.31 × 10^−06^	4	rs2736982	*DSPP*	0.326	0.220	2.09 × 10^−06^
5	rs7736605	*WDR70*	37642044	A	0.059	0.775	0.163	0.051	2.72 × 10^−06^	5	rs7736605	*WDR70*	0.059	0.782	2.26 × 10^−06^
6	rs10499212	intergenic	140027475	T	0.100	0.642	0.136	0.051	3.11 × 10^−06^	14	rs7149414	intergenic	0.133	0.382	2.31 × 10^−06^
6	rs4896151	*LINC00271*	135829796	T	0.141	0.398	0.085	0.049	3.93 × 10^−06^	1	rs11164689	intergenic	0.065	0.500	3.09 × 10^−06^
1	rs11164689	intergenic	103603452	T	0.065	0.491	0.106	0.049	4.32 × 10^−06^	6	rs4896151	*LINC00271*	0.141	0.402	3.42 × 10^−06^
6	rs1547079	*AHI1*	135749202	C	0.177	0.389	0.085	0.047	5.91 × 10^−06^	6	rs1547079	*AHI1*	0.177	0.634	4.69 × 10^−06^
										6	rs9494274	*LINC00271*	0.234	0.394	7.95 × 10^−06^

Chr: Chromosome. SNP: Single nucleotide polymorphism. ^1^: Model 1, unadjusted general linear model (GLM). SNPs were tested in an additive model (0, 1, or 2 minor alleles). ^2^: Model 2, GLM adjusted for sex, age, and diabetes. Beta: Indicates the regression coefficients per one minor allele. SE: Standard error of Beta. Beta ^1^: indicates the regression coefficients for the unadjusted general linear model. Beta ^2^: indicates the regression coefficients for the adjusted general linear model for sex, age, and diabetes. R^2^: Determination coefficient. ^3^: Base position in the chromosome (Homo Sapiens GRCh37.p13 genome build used in Illumina HumanOmniExpress-24 BeadChip). SNPs with *p*-values < 1 × 10^−5^ are listed (n = 426 subjects analyzed).

**Table 3:** Top-ranked SNPs in the GWAS for docosahexaenoic fatty acid concentrations (%) in the whole population.

Unadjusted ^1^										Adjusted ^2^					
Chr	SNP	Gene	Position ^3^	Allele	MAF	Beta ^1^	SE ^1^	R^2^ ^(1)^	*p* ^1^	Chr	SNP	Gene	MAF	Beta ^2^	*p* ^2^
11	rs174547	*FADS1*	61570783	C	0.298	−0.122	0.017	0.106	6.12 × 10^−12^	11	rs174547	*FADS1*	0.298	−0.121	1.28 × 10^−11^
11	rs174550	*FADS1*	61571478	C	0.298	−0.121	0.017	0.105	7.35 × 10^−12^	11	rs174550	*FADS1*	0.298	−0.121	1.50 × 10^−11^
11	rs174546	*FADS1*	61569830	T	0.298	−0.121	0.017	0.104	8.24 × 10^−12^	11	rs174546	*FADS1*	0.298	−0.120	1.66 × 10^−11^
11	rs1535	*FADS2*	61597972	G	0.322	−0.118	0.017	0.103	1.29 × 10^−11^	11	rs1535	*FADS2*	0.322	−0.117	2.87 × 10^−11^
11	rs174583	*FADS2*	61609750	T	0.369	−0.115	0.017	0.102	1.54 × 10^−11^	11	rs174583	*FADS2*	0.369	−0.114	3.34 × 10^−11^
11	rs174576	*FADS2*	61603510	A	0.363	−0.115	0.017	0.101	2.11 × 10^−11^	11	rs174576	*FADS2*	0.363	−0.114	4.42 × 10^−11^
11	rs174577	*FADS2*	61604814	A	0.392	−0.109	0.017	0.092	1.66 × 10^−10^	11	rs174538	*FEN1*	0.282	−0.117	3.40 × 10^−10^
11	rs174538	*FEN1*	61560081	A	0.282	−0.117	0.018	0.092	1.77 × 10^−10^	11	rs174577	*FADS2*	0.392	−0.109	3.47 × 10^−10^
11	rs174535	*MYRF*	61551356	C	0.340	−0.111	0.017	0.091	1.91 × 10^−10^	11	rs174535	*MYRF*	0.340	−0.111	3.89 × 10^−10^
11	rs102275	*TMEM258*	61557803	C	0.493	−0.108	0.017	0.088	4.14 × 10^−10^	11	rs102275	*TMEM258*	0.493	−0.108	8.79 × 10^−10^
11	rs108499	*MYRF*	61547237	T	0.288	−0.112	0.018	0.087	5.76 × 10^−10^	11	rs108499	*MYRF*	0.288	−0.111	1.22 × 10^−09^
11	rs174532	*MYRF*	61548874	A	0.100	0.089	0.017	0.061	2.19 × 10^−07^	11	rs174532	*MYRF*	0.100	0.088	3.43 × 10^−07^
6	rs2092556	*LINC00271*	135899344	C	0.188	0.154	0.031	0.055	1.13 × 10^−06^	6	rs2092556	*LINC00271*	0.188	0.154	1.24 × 10^−06^
6	rs10499212	intergenic	140027475	T	0.100	0.239	0.050	0.052	2.27 × 10^−06^	4	rs2736982	*DSPP*	0.326	0.081	2.25 × 10^−06^
1	rs3001080	intergenic	163434750	G	0.426	−0.082	0.017	0.050	2.90 × 10^−06^	6	rs10499212	intergenic	0.100	0.238	2.64 × 10^−06^
6	rs4896151	*LINC00271*	135829796	T	0.141	0.148	0.031	0.050	2.90 × 10^−06^	6	rs4896151	*LINC00271*	0.141	0.149	3.04 × 10^−06^
4	rs2736982	*DSPP*	88534235	A	0.326	0.079	0.017	0.050	3.13 × 10^−06^	1	rs3001080	intergenic	0.426	−0.081	3.57 × 10^−06^
1	rs16856236	*DISC1*	232160058	G	0.062	0.188	0.040	0.050	3.38 × 10^−06^	6	rs1547079	*AHI1*	0.177	0.146	3.91 × 10^−06^
6	rs1547079	*AHI1*	135749202	C	0.177	0.146	0.031	0.049	3.85 × 10^−06^	1	rs16856236	*DISC1*	0.062	0.186	4.01 × 10^−06^
5	rs7736605	*WDR70*	37642044	A	0.059	0.280	0.060	0.049	3.86 × 10^−06^	5	rs7736605	*WDR70*	0.059	0.281	4.05 × 10^−06^
6	rs9494274	*LINC00271*	135880480	G	0.234	0.135	0.029	0.048	5.55 × 10^−06^	5	rs9312754	*CTNND2*	0.261	0.123	5.24 × 10^−06^
5	rs9312754	*CTNND2*	11088028	C	0.261	0.122	0.027	0.047	6.18 × 10^−06^	6	rs9494274	*LINC00271*	0.234	0.135	6.35 × 10^−06^
18	rs165155	intergenic	75341905	C	0.163	−0.110	0.024	0.047	6.53 × 10^−06^	18	rs165155	intergenic	0.163	−0.109	8.42 × 10^−06^
10	rs16911516	intergenic	59751257	C	0.086	−0.337	0.074	0.047	7.04 × 10^−06^	10	rs16911516	intergenic	0.086	−0.336	8.92 × 10^−06^
										1	rs11164689	intergenic	0.065	0.175	9.18 × 10^−06^

Chr: Chromosome. SNP: Single nucleotide polymorphism. ^1^: Model 1, unadjusted general linear model (GLM). SNPs were tested in an additive model (0, 1, or 2 minor alleles). ^2^: Model 2, GLM adjusted for sex, age and diabetes. Beta: Indicates the regression coefficients per one minor allele. SE: Standard error of beta. Beta ^1^: indicates the regression coefficients for the unadjusted general linear model. Beta ^2^: indicates the regression coefficients for the adjusted general linear model for sex, age, and diabetes. R^2^: Determination coefficient. ^3^: Base position in the chromosome (Homo Sapiens GRCh37.p13 genome build used in Illumina HumanOmniExpress-24 BeadChip). SNPs with *p* < 5 × 10^−5^ are listed.

**Table 4:** Top-ranked SNPs in the RWAS for the *FADS1*/*FADS2*/*FADS3* region in determining serum linoleic fatty acid concentrations (%) in the whole population.

Adjusted ^1^							
Chr	SNP	Gene	Position^2^	Alleles	MAF	Beta	*p*
11	rs174611	*FADS2*	61627881	C	0.116	0.679	4.86 × 10^−04^
11	rs174618	*FADS2*	61629322	C	0.349	0.550	1.21 × 10^−03^
11	rs174547	*FADS1*	61570783	C	0.298	0.527	2.78 × 10^−03^
11	rs174583	*FADS2*	61609750	T	0.369	0.507	2.88 × 10^−03^
11	rs174546	*FADS1*	61569830	T	0.298	0.521	3.19 × 10^−03^
11	rs174550	*FADS1*	61571478	C	0.298	0.512	3.82 × 10^−03^
11	rs174576	*FADS2*	61603510	A	0.363	0.493	3.83 × 10^−03^
11	rs174577	*FADS2*	61604814	A	0.392	0.491	3.89 × 10^−03^
11	rs174450	*FADS3*	61641542	T	0.435	0.481	5.15 × 10^−03^
11	rs1535	*FADS2*	61597972	G	0.322	0.482	5.49 × 10^−03^
11	rs17156442	*FADS2*	61614023	T	0.098	0.976	8.04 × 10^−03^
11	rs174455	*FADS3*	61656117	A	0.407	0.401	1.93 × 10^−02^
11	rs1000778	*FADS3*	61655305	A	0.477	0.329	7.15 × 10^−02^
11	rs174570	*FADS2*	61597212	T	0.228	0.219	3.84 × 10^−01^
11	rs2727270	*FADS2*	61603237	T	0.163	0.236	4.08 × 10^−01^
11	rs482548	*FADS2*	61633182	T	0.030	0.216	4.19 × 10^−01^
11	rs17764288	*FADS2*	61633736	A	0.003	0.520	5.00 × 10^−01^
11	rs12807005	*FADS2*	61591059	A	0.006	0.357	6.30 × 10^−01^
11	rs2851682	*FADS2*	61616012	G	0.149	0.133	6.75 × 10^−01^
11	rs498793	*FADS2*	61624705	T	0.313	−0.048	7.77 × 10^−01^
11	rs7942717	*FADS3*	61647288	G	0.097	0.040	8.83 × 10^−01^

Chr: Chromosome. SNP: Single nucleotide polymorphism. ^1^: Model 2, general linear model (GLM) adjusted for sex, age, and diabetes: SNPs were tested in an additive model (0, 1, or 2 minor alleles). Beta: Indicates the regression coefficients per one minor allele for the adjusted general linear model for sex and age. MAF: Minor allele frequency. ^2^: Base position in the chromosome (Homo Sapiens GRCh37.p13 genome build used in Illumina HumanOmniExpress-24 BeadChip). SNPs with *p* < 5 × 10^-5^ are listed.

## Supplementary Materials

The following are available online at https://www.mdpi.com/2072-6643/12/2/310/s1: Supplemental Figure S1: Conversion flow of omega-3 and omega-6 fatty acids, respectively modulated by *FADS1*/*FASD2* and *ELOVL2*/*ELOVL5* enzymes, from linoleic acid to β-docosapentaenoic acid and from α-linolenic acid to docosahexaenoic acid; Supplemental Table S1: Quantitative 17-item questionnaire for Adherence to Mediterranean diet; Supplemental Table S2: Other medications of the study population by sex; Supplemental Figure S2: Q–Q plot for the GWAS on serum omega-3 fatty acid concentrations (%) in the whole population; Supplemental Table S3: Genome-wide interaction study with adherence to Mediterranean diet in determining serum omega-3 fatty acid concentrations (%) in the whole population. Top-ranked SNP*diet interaction terms; Supplemental Figure S3: Q–Q plot for the genome-wide interaction study with adherence to Mediterranean diet in determining serum omega-3 fatty acid concentrations (%) in the whole population; Supplemental Figure S4: Q–Q plot for the GWAS on total docosahexaenoic fatty acid concentrations (%) in the whole population; Supplemental Figure S5. Manhattan plot for the GWAs results for the serum omega-6 fatty acids (%) in the whole population; Supplemental Figure S6. Q–Q plot for the GWAS on serum omega-6 fatty acid concentrations (%) in the whole population; Supplemental Table S4: Top-ranked SNPs in the GWAS for serum omega-6 fatty acids concentrations (%) in the whole population; Supplemental Figure S7: Manhattan plot for the GWAS results for the linoleic fatty acids (%) in the whole population; Supplemental Figure S8: Q–Q plot for the GWAS on linoleic fatty acid concentrations (%) in the whole population; Supplemental Table S5: Top-ranked SNPs in the GWAS for linoleic fatty acids concentrations (%) in the whole population; Supplemental Figure S9. Manhattan plot for the genome-wide interaction with adherence to Mediterranean diet in determining polyunsaturated fatty acids (%) in the whole population; Supplemental Table S6: Top-ranked SNPs in the GWAS for the serum omega-6/omega-3 PUFA ratio in the whole population; Supplemental Table S7: SNPs in the RWAS for the *FADS1*/*FADS2*/*FADS3* region in determining serum omega-3 fatty acid concentrations (%) in the whole population; Supplemental Table S8: SNPs in the RWAS for the *FADS1*/*FADS2*/*FADS3* region in determining serum docosahexaenoic fatty acid concentrations (%) in the whole population; Supplemental Table S9: SNPs in the RWAS for the *FADS1*/*FADS2*/*FADS3* region in determining serum omega-6 fatty acid concentrations (%) in the whole population; Supplemental Table S10: SNPs in the RWAS for the *ELOVL2*/*ELOVL5* region in determining serum omega-3 fatty acid concentrations (%) in the whole population; Supplemental Table S11: SNPs in the RWAS for the *ELOVL2*/*ELOVL5* region in determining serum omega-6 fatty acid concentrations (%) in the whole population; Supplemental Table S12: SNPs in the RWAS for the *ELOVL2*/*ELOVL5* region in determining linoleic fatty acid concentrations (%) in the whole population Supplemental Figure S10. Means of serum omega-6/omega-3 PUFA ratio depending on the *FADS1*-rs174547 genotype in the whole population; Supplemental Figure S11. Manhattan plot for the GWAs interaction results between the top-ranked SNPs and the adherence to the Mediterranean diet in determining serum omega-6/omega-3 PUFA ratio in the whole population.
