# Supplementary material for: Genome-Wide Association Study for Serum Omega-3 and Omega-6 Polyunsaturated Fatty Acids: Exploratory Analysis of the Sex-Specific Effects and Dietary Modulation in Mediterranean Subjects with Metabolic Syndrome
Source: Nutrients. 2020 Jan 24;12(2):310. doi: 10.3390/nu12020310 (PMC7071282; doi:10.3390/nu12020310)
Supplement: Supplementary file 1 [file nutrients-12-00310-s001.pdf]

ONLINE SUPPORTING MATERIAL

Genome-Wide Association Study for Serum Omega-3 and Omega-6 Polyunsaturated Fatty Acids: Exploratory Analysis of the Sex-Specific Effects and Dietary Modulation in Mediterranean Subjects with Metabolic Syndrome

Oscar Coltell, Jose V. Sorlí, Eva M. Asensio, Rocío Barragán, José I. González, Ignacio M. Giménez-Alba, Vicente Zanón-Moreno, Ramon Estruch, Judith B Ramírez-Sabio, Eva C. Pascual, Carolina Ortega-Azorín, Jose M Ordovas and Dolores Corella

Supplemental Figure 1 2
Supplemental Table 1.......... 3
Supplemental Table 2.......... 4
Supplemental Figure 2. 5
Supplemental Table 3.......... 6
Supplemental Figure 3 7
Supplemental Figure 4 8
Supplemental Figure 5 9
Supplemental Figure 6 10
Supplemental Table 4.......... 11
Supplemental Figure 7 12
Supplemental Figure 8 13
Supplemental Table 5.......... 14
Supplemental Figure 9 15
Supplemental Table 6.......... 16
Supplemental Table 7.......... 17
Supplemental Table 8.......... 18
Supplemental Table 9.......... 19
Supplemental Table 10.......... 20
Supplemental Table 11.......... 21
Supplemental Table 12.......... 22
Supplemental Figure 10 23
Supplemental Figure 11 24
References 25

Supplemental Figure 1. Conversion flow of Omega-3 and Omega-6 fatty acids, respectively modulated by FADS1/2 and ELOVL2/5 enzymes, from linoleic acid to β-docosapentaenoic fatty acid and from α-linolenic fatty acid to docosahexaenoic acid. Adapted from [1-3].

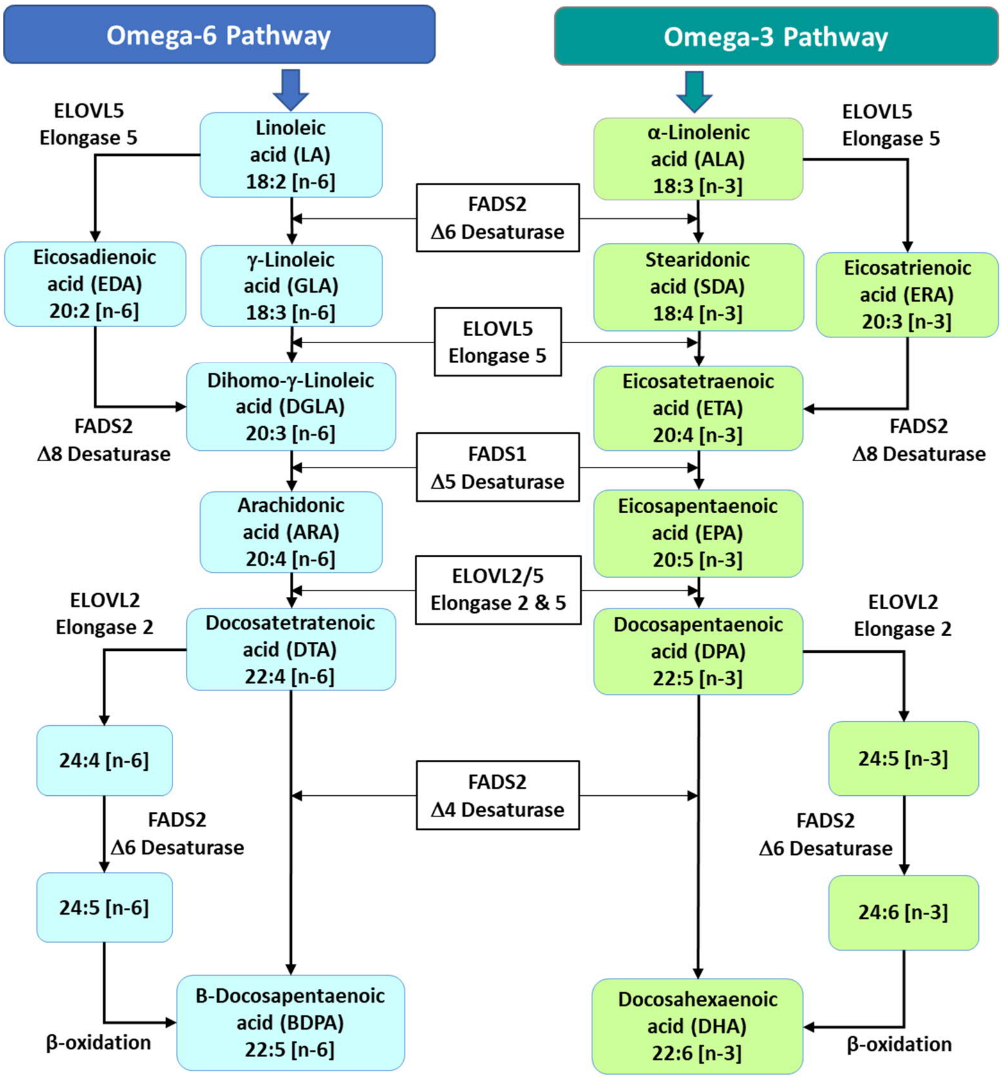

Supplemental Table 1. Quantitative 17-item questionnaire for Adherence to Mediterranean diet.

	**Food items and frequency of consumption**	**Criteria for 1 point**[1]
1.	Do you use olive oil as main culinary fat?	Yes
2.	How many vegetable servings do you consume per day? (1 serving = 200g [consider side dish as half serving])	≥2 (≥1 portion raw or as salad)
3.	How many fruit units (including natural fruit juices) do you consume per day?	≥3
4.	How many servings of red meat, hamburger or meat products (ham, sausage, etc.) do you consume per week? (1 serving = 100–150g)	<1
5.	How many servings of butter, margarine, or cream do you consume per week? (1 serving = 12g)	<1
6.	How many sweetened beverages (soft drinks, cola, bitter, juices without added sugars) do you drink per week?	<1
7.	How many servings of legumes do you consume per week? (1 serving = 150g)	≥3
8.	How many servings of fish or shellfish do you consume per week? (1 serving = 100-150g of fish or 4-5 units or 200g of shellfish)	≥3
9.	How many times per week do you consume pastries, such as cookies, custard, sweets or cakes?	<3
10.	How many servings of nuts (including peanuts) do you consume per week? (1 serving = 30g)	≥1
11.	Do you preferentially consume chicken, turkey, or rabbit meat instead of veal, pork, hamburger, or sausage?	Yes
12.	How many times per week do you consume vegetables, pasta, rice, or other dishes seasoned with *sofrito* (sauce made with tomato and onion, leek, or garlic and simmered with olive oil)?	≥2
13.	Do you add sugar to the coffee or tea?	No/use artificial sweeteners
14.	How many servings of white bread do you consume per day? (1 serving = 75g)	<1
15.	How many servings of cereals and whole grains (bread, rice, pasta) do you consume per week?	≥5
16.	How many servings of white bread, rice and/or pasta do you consume per week?	<3
17.	How much wine do you drink per week?	2-3 glasses/day for men and 1-2 glasses/day for women

[1] ‘0’ points if these criteria are not met.

Supplemental Table 2. Other medications[1] of the study population by sex.

	Total (n=426)	Men (n=187)	Women (n=239)	p
Other antidiabetic drugs[2]: n, %	121 (28.4)	56 (29.9)	65 (27.2)	0.532
Treatments for pain and fever: n, %	88 (20.7)	25 (13.4)	63 (26.4)	0.001
Anti-platelet drugs: n, %	50 (1.7)	23 (12.3)	27 (11.3)	0.750
Tranquilizers-sedative-hypnotic: n, %	130 (30.5)	22 (11.8)	108 (45.2)	<0.001
Vitamins and minerals: n, %	51 (12.0)	11 (5.9)	40 (16.7)	0.001
Cardio protective drugs: n, %	25 (5.9)	11 (5.9)	14 (5.9)	0.991

Values are numbers and %. p: p-value for the comparisons of % between men and women; [1]: Self-reported medications taken during the last month; [2]: Other antidiabetic medications include treatments used as a single oral agent or combined with one or more antidiabetic drugs.

Supplemental Figure 2. Q-Q plot for the GWAS on serum omega-3 fatty acid concentrations (%) in the whole population.

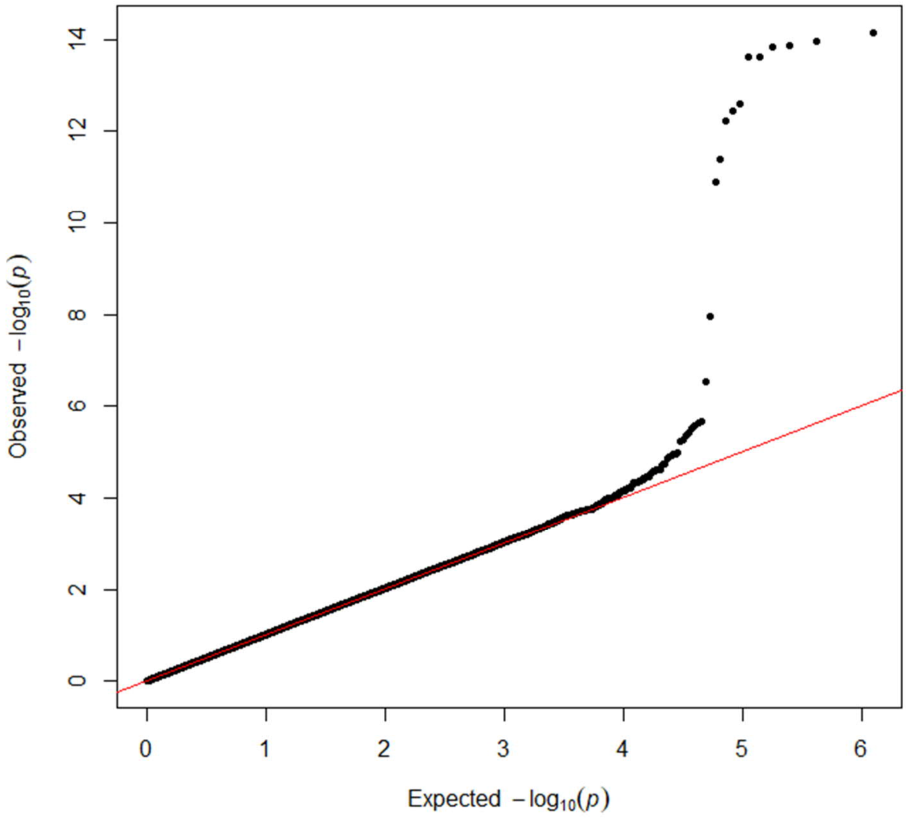

Supplemental Table 3. Genome-wide interaction study with adherence to Mediterranean diet in determining serum omega-3 fatty acid concentrations (%) in the whole population. Top-ranked SNP*diet interaction terms.

Chr	SNP	Beta[1]	SE[1]	Beta[2]	SE[2]	p-Gene*AdhMedDiet Interaction[3]	Alleles	MAF	Gene
6	rs3798890	0.199	0.064	-0.292	0.070	2.15×10^{-07}	A	0.193091	ME1
6	rs6917851	0.203	0.064	-0.286	0.070	2.17×10^{-07}	A	0.192093	ME1
6	rs1180204	0.248	0.064	-0.211	0.065	4.98×10^{-07}	A	0.398163	ME1
6	rs12191369	0.197	0.066	-0.290	0.072	5.46×10^{-07}	C	0.188498	ME1
6	rs12203860	0.200	0.063	-0.264	0.069	7.05×10^{-07}	G	0.279952	intergenic
6	rs6454330	0.187	0.064	-0.278	0.069	7.67×10^{-07}	C	0.217452	intergenic
3	rs6781156	0.180	0.061	-0.262	0.067	1.03×10^{-06}	A	0.26877	intergenic
6	rs1180241	0.306	0.067	-0.180	0.074	1.07×10^{-06}	A	0.184704	ME1
1	rs4661702	0.192	0.066	-0.279	0.071	1.10×10^{-06}	G	0.247804	intergenic
3	rs7641425	0.180	0.061	-0.271	0.070	1.15×10^{-06}	T	0.296326	intergenic
6	rs13219666	0.294	0.065	-0.185	0.074	1.22×10^{-06}	C	0.14357	intergenic
6	rs1180237	0.246	0.064	-0.192	0.065	1.73×10^{-06}	C	0.433307	ME1
6	rs1180187	0.255	0.064	-0.187	0.067	2.12×10^{-06}	A	0.46845	ME1
6	rs983087	0.307	0.065	-0.151	0.072	2.41×10^{-06}	C	0.226438	ME1
6	rs9444043	0.236	0.064	-0.193	0.067	3.72×10^{-06}	A	0.495008	intergenic
2	rs16866554	0.268	0.118	-0.533	0.127	3.84×10^{-06}	A	0.135184	CCDC141
13	rs765061	0.277	0.089	-0.325	0.097	5.00×10^{-06}	A	0.166534	intergenic
6	rs1535588	0.203	0.063	-0.224	0.069	5.24×10^{-06}	C	0.315895	ME1
6	rs1144180	0.269	0.068	-0.201	0.078	5.88×10^{-06}	G	0.200879	ME1
6	rs1170485	0.208	0.064	-0.220	0.070	6.08×10^{-06}	T	0.315296	ME1
3	rs2971365	0.265	0.099	-0.347	0.093	7.13×10^{-06}	C	0.313898	intergenic
8	rs7013384	0.633	0.150	-0.383	0.171	8.10×10^{-06}	A	0.186102	intergenic
8	rs6583593	0.636	0.154	-0.386	0.171	8.66×10^{-06}	T	0.148762	intergenic
6	rs1180195	0.253	0.063	-0.150	0.065	9.03×10^{-06}	T	0.449481	ME1
20	rs11699833	0.247	0.065	-0.189	0.074	9.06×10^{-06}	A	0.251398	intergenic
6	rs1180192	0.312	0.067	-0.137	0.076	9.41×10^{-06}	T	0.205871	ME1

[1] SNP: Single Nucleotide Polymorphisms. Only top-ranked SNPs with P-value < 1×10^{-5} for SNP*diet interaction terms in the whole population (n=426) are listed. A 17-item screening questionnaire was used for assessing adherence to Mediterranean diet, defining two groups based on the sample median (8 points): Low adherence to MedDiet (0–8 points), and High adherence to MedDiet (9–17 points). Chr: Chromosome. Beta: Indicates the regression coefficients per one minor allele. SE: Standard error. MAF: Minor allele frequency. Beta[1]: indicates the regression coefficients for the low adherence to Mediterranean diet strata (n=248). Beta[2]: indicates the regression coefficients for the high adherence to Mediterranean diet strata (n=178). [3] P-value obtained for the interaction term SNP*AdhMedDiet in the corresponding hierarchical GLM regression model including the main effects.

Supplemental Figure 3. Q-Q plot for the genome-wide interaction study with adherence to Mediterranean diet in determining serum omega-3 fatty acid concentrations (%) in the whole population.

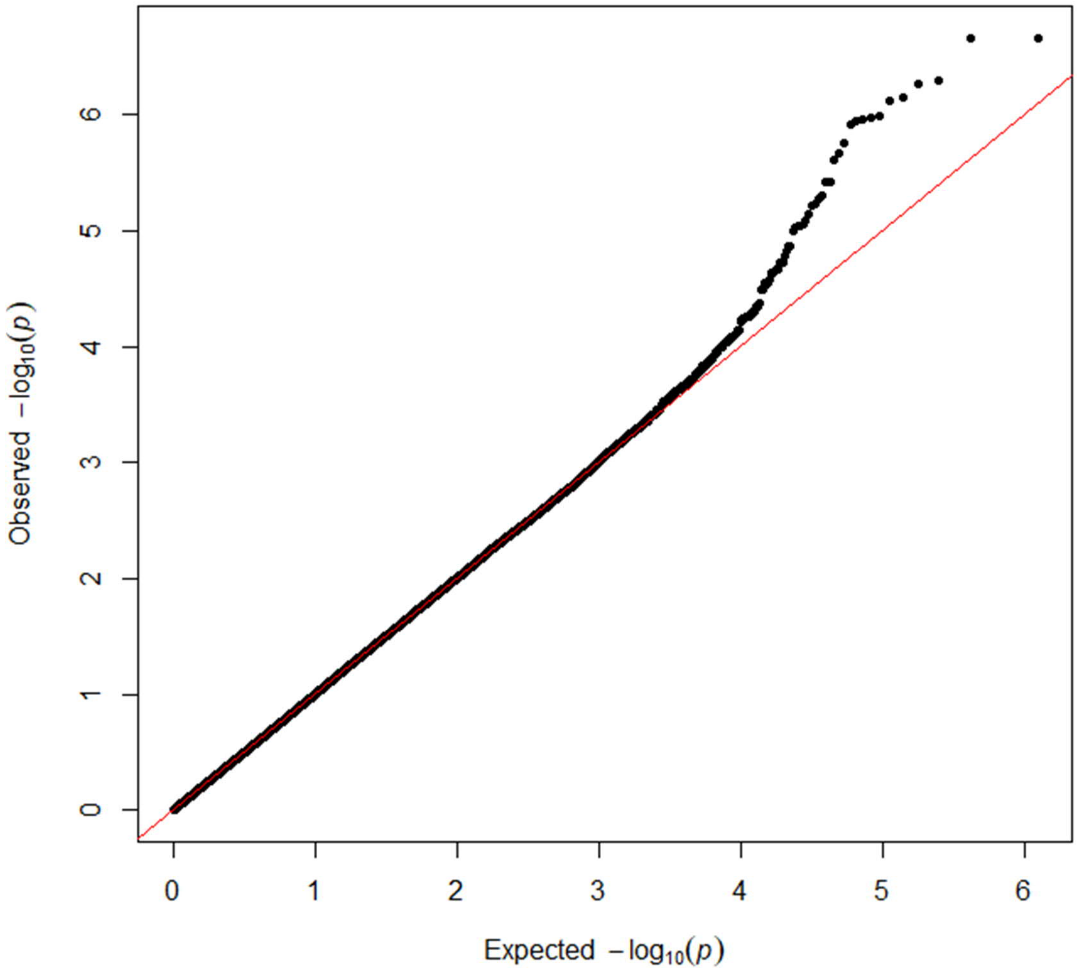

Supplemental Figure 4. Q-Q plot for the GWAS on total docosahexaenoic fatty acid concentrations (%) in the whole population.

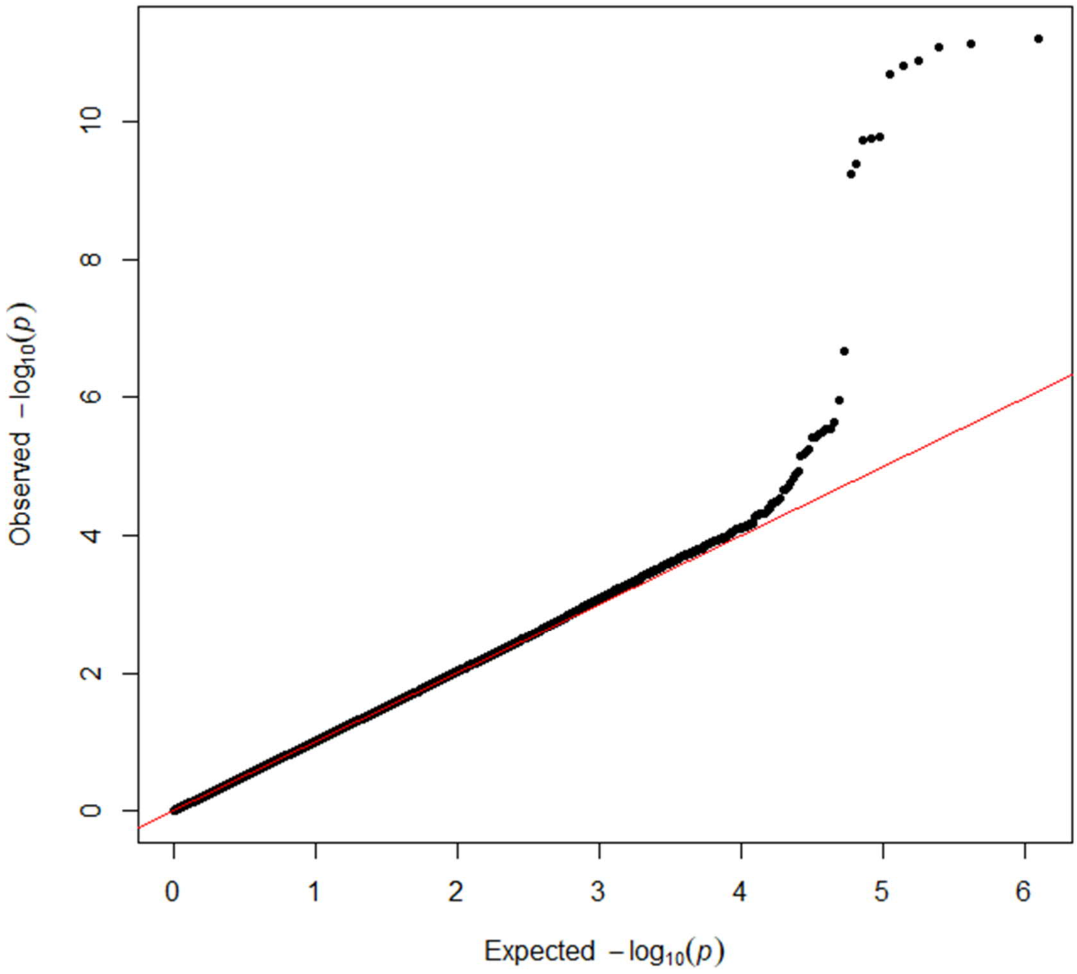

Supplemental Figure 5. Manhattan plot for the GWAs results for the serum omega-6 fatty acid (%) in the whole population. Associations were obtained from the genetic additive model and p-values were expressed as $-\log_{10}(p\text{-value})$. The red line represents the threshold for GWAS statistical significance ($-\log_{10}(5 \times 10^{-8})$). The blue line represents the threshold for suggestive GWAS significance ($-\log_{10}(1 \times 10^{-5})$).

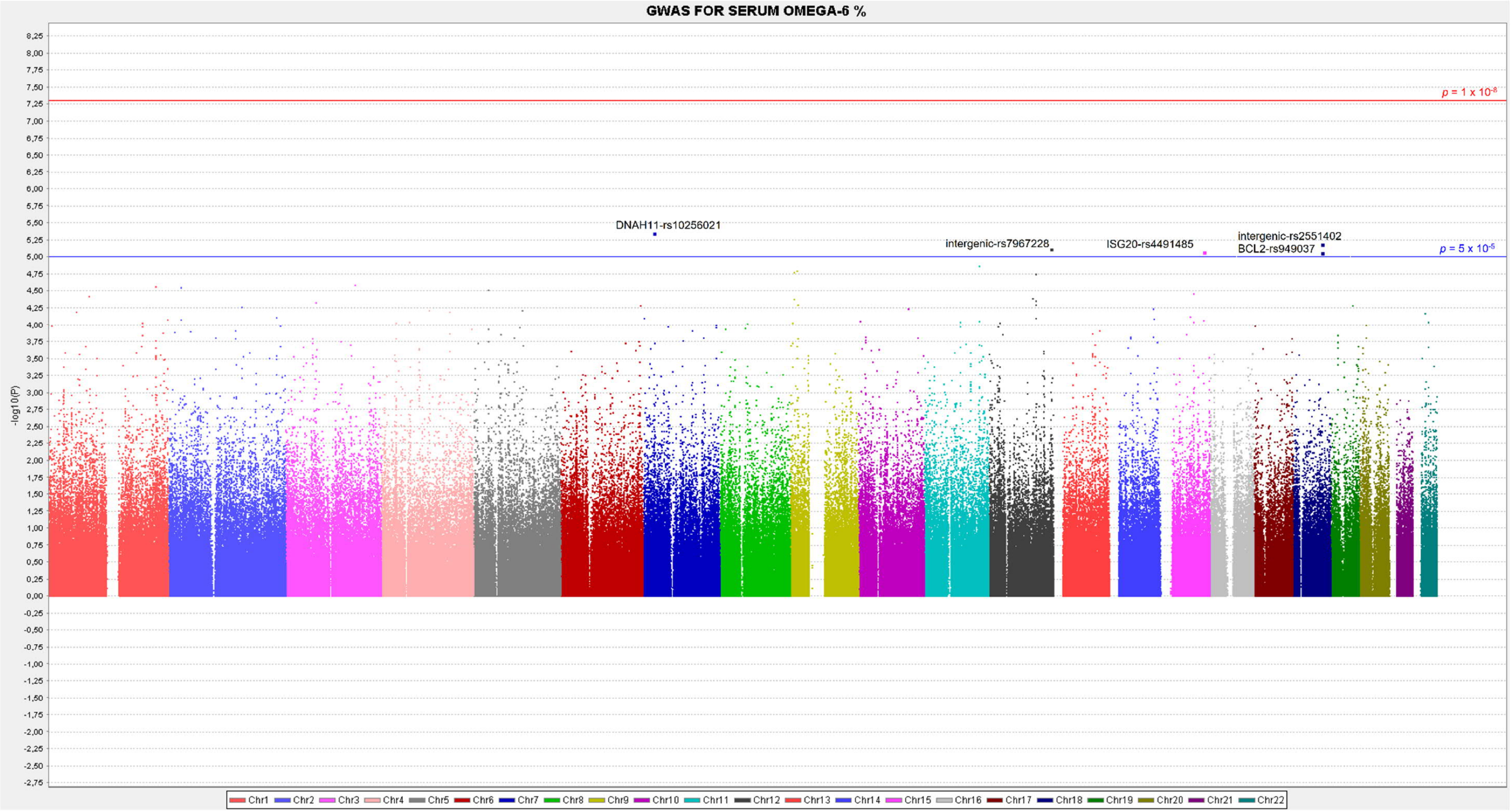

Supplemental Figure 6. Q-Q plot for the GWAS on serum omega-6 fatty acid concentrations (%) in the whole population.

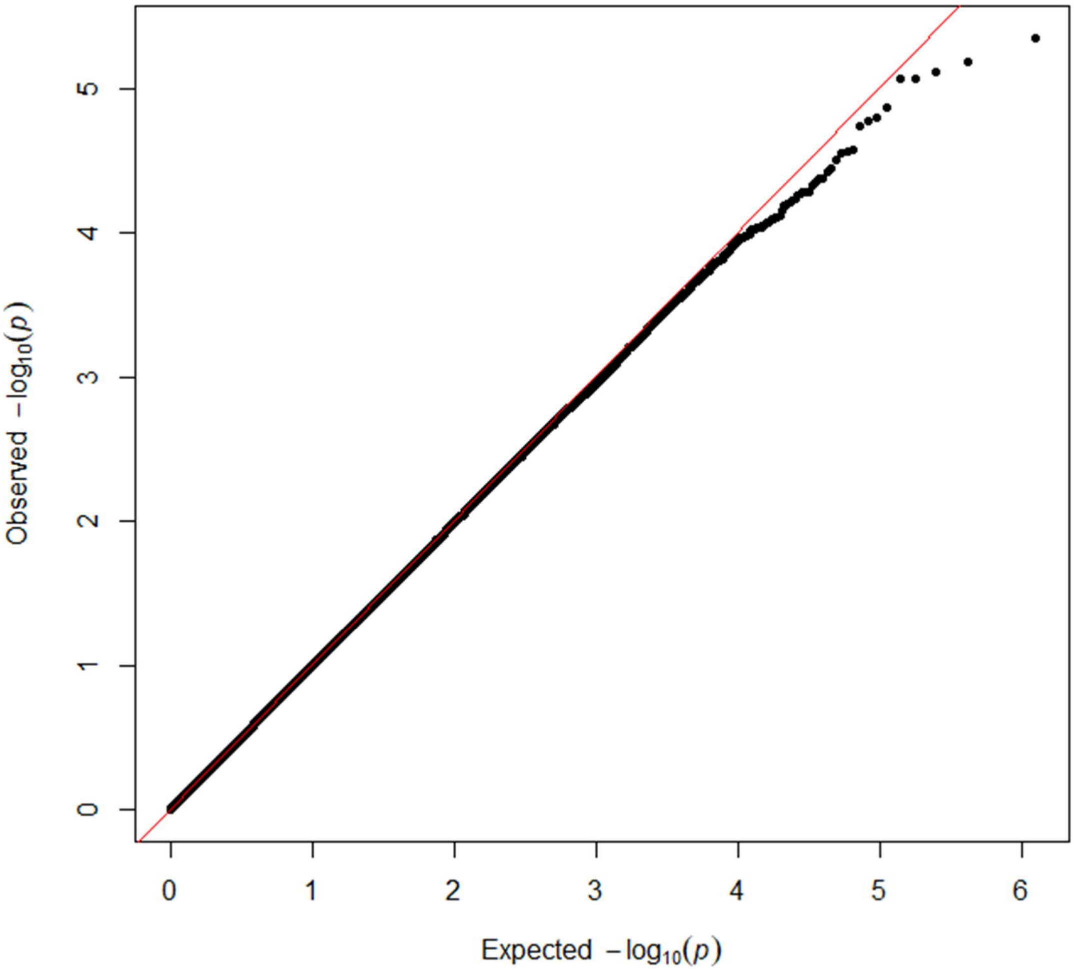

Supplemental Table 4. Top-ranked SNPs in the GWAS for serum omega-6 fatty acid concentrations (%) in the whole population.

Unadjusted[1]									
Chr	SNP	Gene	Position[3]	Alleles	MAF	Beta[1]	SE[1]	$R^{2(1)}$	p[1]
7	rs10256021	DNAH11	21801878	G	0.355	-0.706	0.152	0.049	4.42×10^{-06}
18	rs2551402	intergenic	60990022	C	0.456	0.664	0.145	0.047	6.49×10^{-06}
12	rs7967228	intergenic	127840232	G	0.499	-0.667	0.147	0.046	7.71×10^{-06}
15	rs4491485	ISG20	89180746	G	0.248	-1.133	0.252	0.046	8.57×10^{-06}
18	rs949037	BCL2	60979013	A	0.403	0.648	0.144	0.046	8.65×10^{-06}
11	rs6589490	CADM1	115131900	G	0.350	0.621	0.141	0.044	1.37×10^{-05}
9	rs7047109	intergenic	14555896	C	0.125	-0.833	0.191	0.043	1.60×10^{-05}
9	rs12002314	intergenic	7694465	G	0.177	-0.795	0.183	0.043	1.69×10^{-05}
12	rs252181	intergenic	97283633	T	0.456	0.652	0.150	0.043	1.80×10^{-05}
3	rs4839595	SLC9A9	143163169	G	0.427	-0.618	0.145	0.041	2.63×10^{-05}
1	rs17478205	DISP1	223162746	A	0.391	-0.608	0.143	0.041	2.72×10^{-05}
2	rs6712986	ADCY3	25126046	C	0.203	0.885	0.209	0.041	2.80×10^{-05}
5	rs1921111	intergenic	30870858	A	0.262	0.663	0.157	0.040	3.09×10^{-05}
15	rs430610	intergenic	68198622	G	0.477	-0.591	0.141	0.040	3.51×10^{-05}
1	rs876339	LOC107985396	83340310	C	0.209	-0.697	0.167	0.040	3.76×10^{-05}
12	rs2723934	LOC102724834	91013303	A	0.376	0.639	0.154	0.039	4.15×10^{-05}
9	rs12376595	intergenic	7685789	C	0.179	-0.749	0.181	0.039	4.17×10^{-05}
12	rs10777820	NEDD1	97300540	A	0.487	0.620	0.150	0.039	4.45×10^{-05}
3	rs815710	intergenic	61262149	A	0.323	-0.591	0.144	0.039	4.72×10^{-05}

Adjusted[2]					
Chr	SNP	Gene	MAF	Beta[2]	p[2]
15	rs4491485	ISG20	0.248	-1.214	1.97×10^{-06}
18	rs2551402	intergenic	0.456	0.682	3.52×10^{-06}
18	rs949037	BCL2	0.403	0.667	4.84×10^{-06}
7	rs10256021	DNAH11	0.355	-0.700	5.32×10^{-06}
12	rs7967228	intergenic	0.499	-0.648	1.54×10^{-05}
11	rs6589490	CADM1	0.350	0.617	1.60×10^{-05}
9	rs7047109	intergenic	0.125	-0.831	1.74×10^{-05}
5	rs1921111	intergenic	0.262	0.671	2.61×10^{-05}
3	rs815710	intergenic	0.323	-0.609	2.68×10^{-05}
9	rs12002314	intergenic	0.177	-0.780	2.70×10^{-05}
12	rs252181	intergenic	0.456	0.640	2.73×10^{-05}
2	rs6712986	ADCY3	0.203	0.873	3.86×10^{-05}
14	rs2540875	GPR68	0.187	-0.865	4.00×10^{-05}
3	rs4839595	SLC9A9	0.427	-0.605	4.02×10^{-05}
7	rs6980380	PRKAG2	0.124	0.759	4.34×10^{-05}
2	rs2222760	RND3	0.171	-0.685	4.35×10^{-05}

Chr: Chromosome. SNP Single Nucleotide Polymorphism. [1]: Model 1, unadjusted general linear model (GLM). SNPs were tested in an additive model (0, 1, or 2 minor alleles). [2]: Model 2, GLM adjusted for sex, age and diabetes. MAF: Minor allele frequency. Beta: Indicates the regression coefficients per one minor allele in the corresponding model. SE: Standard error of Beta. R^2: Determination coefficient. [3]: Base position in the chromosome (Homo Sapiens GRCh37.p13 genome build used in Illumina HumanOmniExpress-24 BeadChip).

Supplemental Figure 7. Manhattan plot for the GWAS results for the linoleic fatty acid (%) in the whole population. Associations were obtained from the genetic additive model and *p*-values were expressed as $-\log_{10}(p\text{-value})$. The red line represents the threshold for GWAS statistical significance ($-\log_{10}(5 \times 10^{-8})$). The blue line represents the threshold for suggestive GWAS significance ($-\log_{10}(1 \times 10^{-5})$).

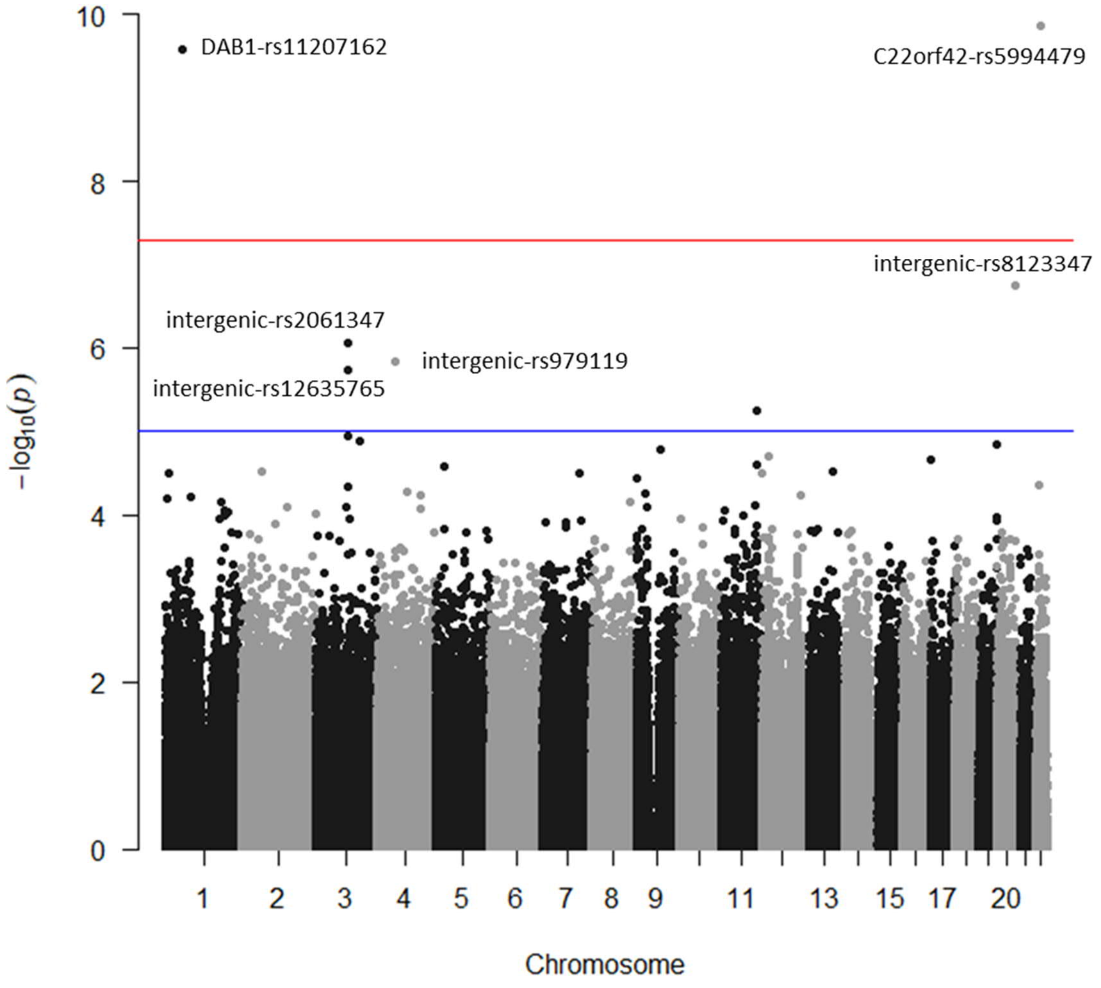

Supplemental Figure 8. Q-Q plot for the GWAS on linoleic fatty acid concentrations (%) in the whole population.

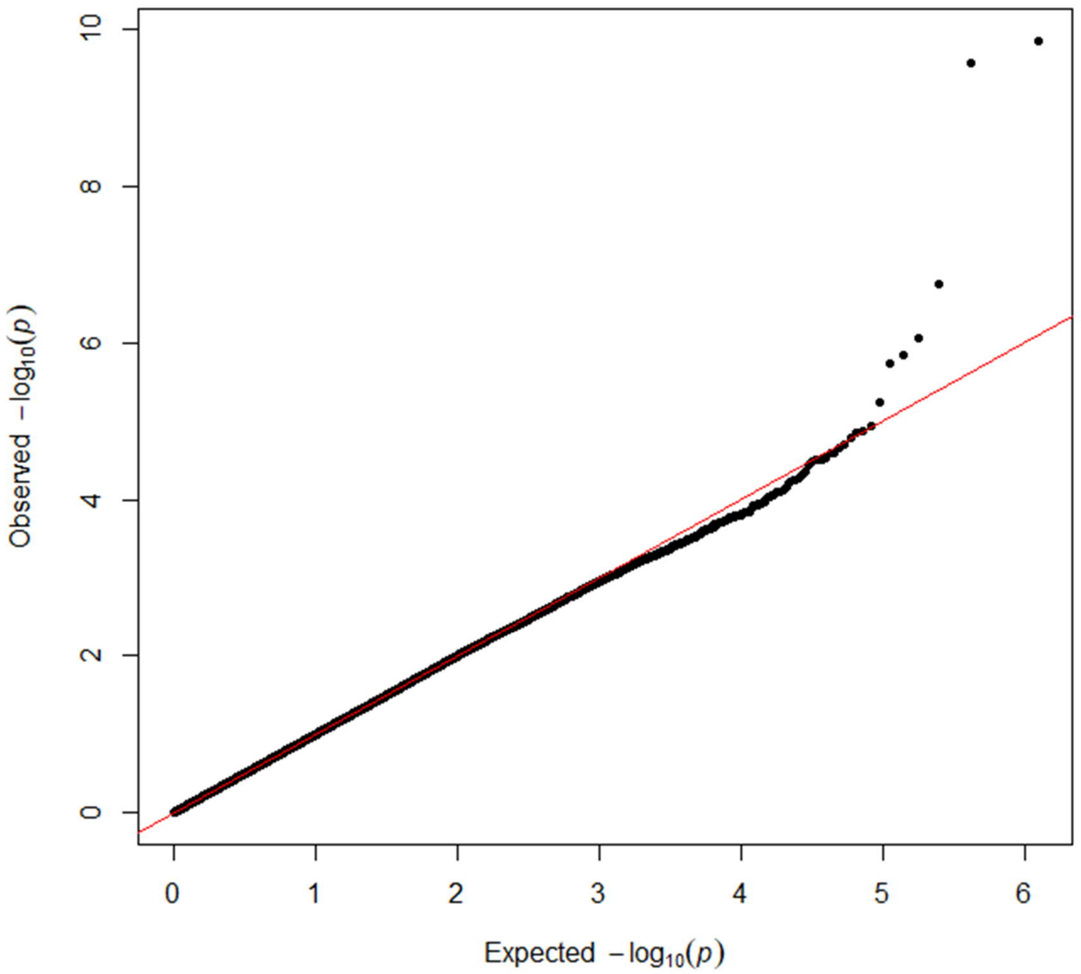

Supplemental Table 5. Top-ranked SNPs in the GWAS for linoleic fatty acid concentrations (%) in the whole population.

Unadjusted[1]									
Chr	SNP	Gene	Position[3]	Alleles	MAF	Beta[1]	SE[1]	$R^{2(1)}$	P[1]
22	rs5994479	C22orf42	32557939	G	0.217	-1.159	0.176	0.095	1.39×10^{-10}
1	rs11207162	DAB1	58526758	T	0.124	1.052	0.162	0.094	2.67×10^{-10}
20	rs8123347	intergenic	59449366	A	0.118	-1.076	0.202	0.079	1.78×10^{-07}
3	rs2061347	intergenic	103492587	A	0.169	-1.147	0.230	0.056	8.60×10^{-07}
4	rs979119	intergenic	58671636	A	0.082	1.675	0.343	0.054	1.46×10^{-06}
3	rs12635765	intergenic	103482015	G	0.104	-1.373	0.284	0.052	1.86×10^{-06}
11	rs6589490	CADM1	115131900	G	0.350	0.733	0.159	0.048	5.72×10^{-06}
3	rs16849343	intergenic	103504420	C	0.306	-0.859	0.194	0.044	1.15×10^{-05}
3	rs4839595	SLC9A9	143163169	G	0.427	-0.724	0.164	0.044	1.32×10^{-05}
19	rs12463137	ZNF773	58011421	T	0.261	0.934	0.213	0.044	1.42×10^{-05}
9	rs273432	intergenic	81866882	G	0.281	-0.817	0.187	0.043	1.62×10^{-05}
12	rs2268861	ST8SIA1	22389474	A	0.373	-0.756	0.175	0.042	1.95×10^{-05}
17	rs178567	RAP1GAP2	2699322	T	0.171	0.735	0.171	0.042	2.21×10^{-05}
11	rs7121254	CADM1	115133557	G	0.388	0.682	0.160	0.041	2.51×10^{-05}
5	rs1921111	intergenic	30870858	A	0.262	0.757	0.178	0.041	2.59×10^{-05}
13	rs7331021	intergenic	95061492	C	0.342	0.836	0.198	0.040	3.02×10^{-05}
2	rs6715876	ACTR2	65486928	C	0.307	0.698	0.166	0.040	3.04×10^{-05}
7	rs11982968	intergenic	121323059	G	0.121	1.149	0.273	0.040	3.14×10^{-05}
12	rs2270033	WNT5B	1743296	C	0.324	-0.758	0.180	0.040	3.14×10^{-05}
1	rs6664362	SLC25A33	9613871	T	0.025	1.366	0.325	0.041	3.20×10^{-05}
9	rs1412254	intergenic	1510230	A	0.125	-0.807	0.193	0.040	3.55×10^{-05}
22	rs5996830	KIAA1671	25465256	C	0.195	-0.871	0.211	0.039	4.30×10^{-05}
3	rs16849322	intergenic	103485884	A	0.101	-1.163	0.282	0.039	4.55×10^{-05}

Adjusted[2]					
Chr	SNP	Gene	MAF	Beta[2]	P[2]
22	rs5994479	C22orf42	0.217	-1.131	5.25×10^{-10}
1	rs11207162	DAB1	0.124	1.031	5.69×10^{-10}
20	rs8123347	intergenic	0.118	-1.059	2.84×10^{-07}
3	rs2061347	intergenic	0.169	-1.182	3.81×10^{-07}
3	rs12635765	intergenic	0.104	-1.375	1.66×10^{-06}
4	rs979119	intergenic	0.082	1.655	2.20×10^{-06}
11	rs6589490	CADM1	0.350	0.741	4.52×10^{-06}
3	rs16849343	intergenic	0.306	-0.877	7.77×10^{-06}
1	rs6664362	SLC25A33	0.025	1.441	1.15×10^{-05}
9	rs273432	intergenic	0.281	-0.826	1.30×10^{-05}
7	rs11982968	intergenic	0.121	1.194	1.46×10^{-05}
5	rs1921111	intergenic	0.262	0.781	1.48×10^{-05}
12	rs2268861	ST8SIA1	0.373	-0.761	1.61×10^{-05}
19	rs12463137	ZNF773	0.261	0.920	1.91×10^{-05}
3	rs4839595	SLC9A9	0.427	-0.711	1.97×10^{-05}
2	rs6715876	ACTR2	0.307	0.710	2.40×10^{-05}
12	rs2270033	WNT5B	0.324	-0.764	2.61×10^{-05}
9	rs1412254	intergenic	0.125	-0.817	2.71×10^{-05}
17	rs178567	RAP1GAP2	0.171	0.725	3.03×10^{-05}
11	rs7938209	SBF2	0.003	1.346	3.04×10^{-05}
11	rs7121254	CADM1	0.388	0.675	3.09×10^{-05}
9	rs34942735	AQP3	0.001	1.375	3.60×10^{-05}
3	rs16849322	intergenic	0.101	-1.170	3.88×10^{-05}
4	rs4699670	intergenic	0.350	-0.734	4.22×10^{-05}
8	rs2385167	TBC1D31	0.077	-1.530	4.87×10^{-05}

Chr: Chromosome. SNP Single Nucleotide Polymorphism. [1]: Model 1, unadjusted general linear model (GLM). SNPs were tested in an additive model (0, 1, or 2 minor alleles). [2]: Model 2, GLM adjusted for sex, age and diabetes. MAF: Minor allele frequency. Beta: Indicates the regression coefficients per one minor allele. SE: Standard error of Beta. Beta[1]: indicates the regression coefficients for the unadjusted general linear model. Beta2: indicates the regression coefficients for the adjusted general linear model for sex, age and diabetes. R^2: Determination coefficient. [3]: Base position in the chromosome (Homo Sapiens GRCh37.p13 genome build used in Illumina HumanOmniExpress-24 BeadChip) ($P<5\times10^{-5}$).

Supplemental Figure 9. Manhattan plot for the genome-wide interaction with adherence to Mediterranean diet in determining polyunsaturated fatty acid (%) in the whole population. Associations were obtained from the genetic additive model and *p*-values were expressed as $-\log_{10}(p\text{-value})$. The red line represents the threshold for GWAS statistical significance ($-\log_{10}(5 \times 10^{-8})$). The blue line represents the threshold for suggestive GWAS significance ($-\log_{10}(1 \times 10^{-5})$).

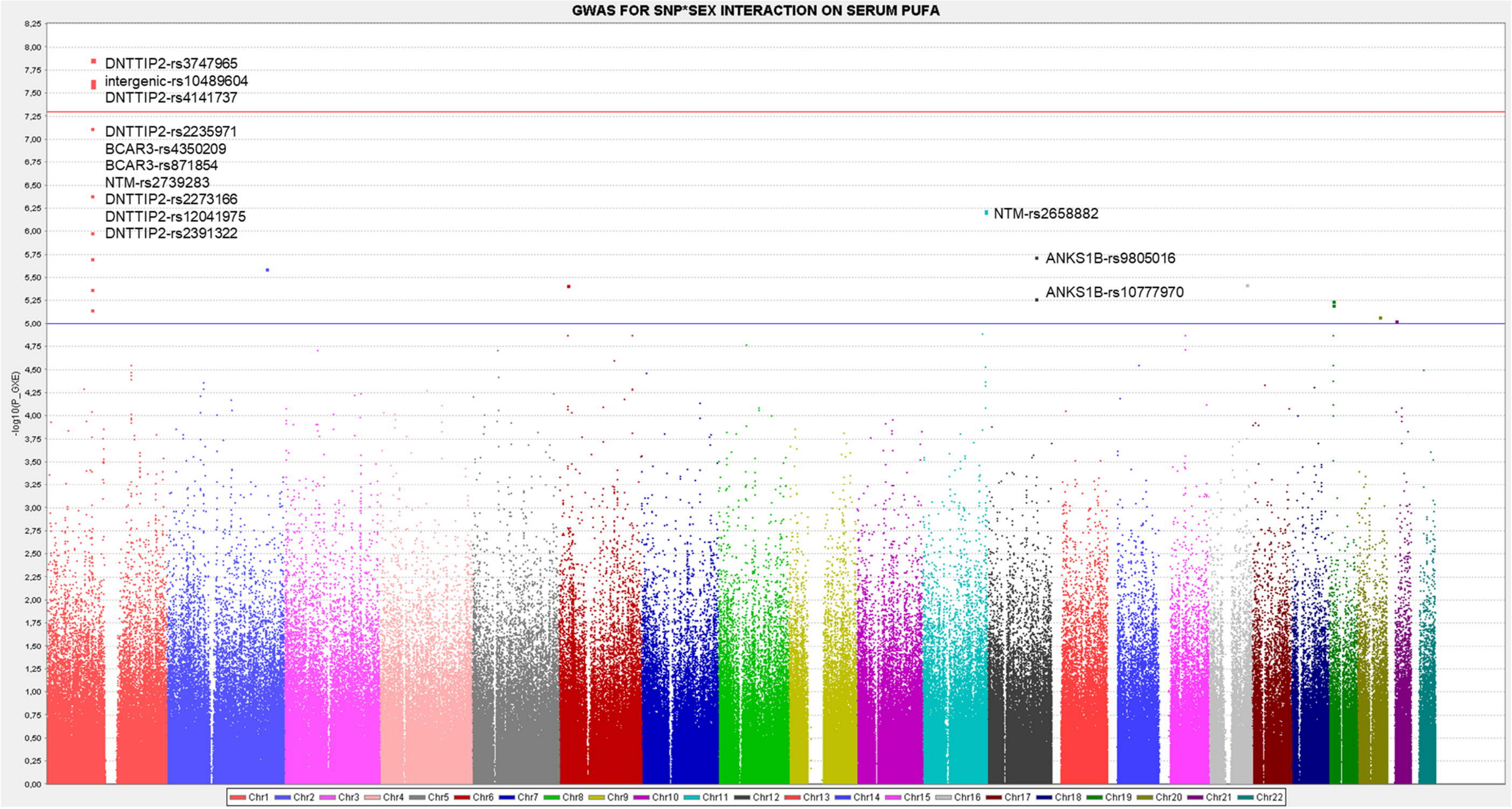

Supplemental Table 6. Top-ranked SNPs in the GWAS for the serum omega-6/omega-3 PUFA ratio in the whole population.

Unadjusted[1]										Adjusted[2]					
Chr	SNP	Gene	Position[3]	Alleles	MAF	Beta[1]	SE[1]	$R^{2(1)}$	P[1]	Chr	SNP	Gene	MAF	Beta[2]	P[2]
11	rs174547	FADS1	61803311	C	0.298	0.042	0.005	0.137	3.91×10^{-15}	11	rs174547	FADS1	0.298	0.041	2.19×10^{-14}
11	rs174583	FADS2	61842278	T	0.369	0.040	0.005	0.136	4.69×10^{-15}	11	rs174583	FADS2	0.369	0.040	2.21×10^{-14}
11	rs174550	FADS1	61804006	C	0.298	0.042	0.005	0.135	6.13×10^{-15}	11	rs174550	FADS1	0.298	0.041	3.49×10^{-14}
11	rs174546	FADS1	61802358	T	0.298	0.042	0.005	0.134	6.83×10^{-15}	11	rs1535	FADS2	0.322	0.040	3.72×10^{-14}
11	rs1535	FADS2	61830500	G	0.322	0.041	0.005	0.134	7.97×10^{-15}	11	rs174546	FADS1	0.298	0.041	3.90×10^{-14}
11	rs174576	FADS2	61836038	A	0.363	0.040	0.005	0.134	8.21×10^{-15}	11	rs174576	FADS2	0.363	0.039	4.13×10^{-14}
11	rs174535	MYRF	61783884	C	0.340	0.040	0.005	0.126	4.99×10^{-14}	11	rs174577	FADS2	0.392	0.038	2.31×10^{-13}
11	rs174577	FADS2	61837342	A	0.392	0.039	0.005	0.126	5.05×10^{-14}	11	rs174535	MYRF	0.340	0.039	2.46×10^{-13}
11	rs102275	TMEM258	61790331	C	0.493	0.039	0.005	0.123	1.12×10^{-13}	11	rs102275	TMEM258	0.493	0.038	4.43×10^{-13}
11	rs174538	FEN1	61792609	A	0.282	0.039	0.005	0.110	2.69×10^{-12}	11	rs174538	FEN1	0.282	0.038	1.41×10^{-11}
11	rs108499	MYRF	61779765	T	0.288	0.038	0.005	0.105	7.31×10^{-12}	11	rs108499	MYRF	0.288	0.037	3.07×10^{-11}
11	rs174532	MYRF	61781402	A	0.100	-0.029	0.005	0.069	3.75×10^{-08}	11	rs174532	MYRF	0.100	-0.029	4.75×10^{-08}
11	rs2727270	FADS2	61835765	T	0.163	0.043	0.009	0.056	7.67×10^{-07}	13	rs2390277	__	0.359	-0.026	3.89×10^{-07}
3	rs7611558	intergenic	30093002	T	0.380	-0.025	0.005	0.057	7.87×10^{-07}	3	rs7611558	__	0.380	-0.024	1.65×10^{-06}
13	rs2390277	intergenic	99406265	A	0.359	-0.025	0.005	0.054	1.27×10^{-06}	11	rs2727270	FADS2	0.163	0.042	1.83×10^{-06}
3	rs4426643	intergenic	30094871	G	0.420	-0.024	0.005	0.054	1.51×10^{-06}	3	rs11715853	__	0.317	-0.025	2.10×10^{-06}
3	rs11715853	intergenic	30122198	G	0.317	-0.025	0.005	0.053	1.57×10^{-06}	3	rs4426643	__	0.420	-0.023	3.17×10^{-06}
10	rs2207778	NRG3	82013458	A	0.113	-0.034	0.007	0.050	3.98×10^{-06}	4	rs2736982	DSPP	0.326	-0.024	4.26×10^{-06}
8	rs7812327	ASPH	61641853	T	0.333	-0.027	0.006	0.047	6.81×10^{-06}	20	rs11908058	CFAP61	0.288	-0.025	6.23×10^{-06}
20	rs11908058	CFAP61	20340192	A	0.288	-0.025	0.006	0.046	8.30×10^{-06}	10	rs2207778	NRG3	0.113	-0.033	7.44×10^{-06}
10	rs10884061	NRG3	82030465	A	0.245	-0.031	0.007	0.046	8.77×10^{-06}	1	rs7528478	LOC107985412	0.471	0.023	8.04×10^{-06}

Chr: Chromosome. SNP Single Nucleotide Polymorphism. [1]: Model 1, unadjusted general linear model (GLM). SNPs were tested in an additive model (0, 1, or 2 minor alleles). [2]: Model 2, GLM adjusted for sex, age and diabetes. MAF: Minor allele frequency. Beta: Indicates the regression coefficients per one minor allele. SE: Standard error of Beta. Beta[1]: indicates the regression coefficients for the unadjusted general linear model. Beta2: indicates the regression coefficients for the adjusted general linear model for sex, age and diabetes. R^2: Determination coefficient. [3]: Base position in the chromosome (Homo Sapiens GRCh37.p13 genome build used in Illumina HumanOmniExpress-24 BeadChip) ($P<5\times10^{-5}$).

Supplemental Table 7. SNPs in the RWAS for the FADS1/FADS2/FADS3 region in determining serum omega-3 fatty acid concentrations (%) in the whole population.

				Adjusted[1]			
Chr	SNP	Gene	Position[2]	Alleles	MAF	Beta	p
11	rs174547	FADS1	61570783	C	0.298	-0.369	1.44×10^{-14}
11	rs174550	FADS1	61571478	C	0.298	-0.367	2.26×10^{-14}
11	rs1535	FADS2	61597972	G	0.322	-0.359	2.55×10^{-14}
11	rs174546	FADS1	61569830	T	0.298	-0.365	2.81×10^{-14}
11	rs174583	FADS2	61609750	T	0.369	-0.349	4.26×10^{-14}
11	rs174576	FADS2	61603510	A	0.363	-0.350	5.00×10^{-14}
11	rs174577	FADS2	61604814	A	0.392	-0.336	4.80×10^{-13}
11	rs2727270	FADS2	61603237	T	0.163	-0.400	3.84×10^{-07}
11	rs174570	FADS2	61597212	T	0.228	-0.295	2.20×10^{-05}
11	rs2851682	FADS2	61616012	G	0.149	-0.339	1.18×10^{-04}
11	rs174611	FADS2	61627881	C	0.116	-0.175	1.35×10^{-03}
11	rs174618	FADS2	61629322	C	0.349	-0.136	4.38×10^{-03}
11	rs174450	FADS3	61641542	T	0.435	-0.122	1.15×10^{-02}
11	rs174455	FADS3	61656117	A	0.407	-0.107	2.65×10^{-02}
11	rs17156442	FADS2	61614023	T	0.098	-0.216	3.68×10^{-02}
11	rs12807005	FADS2	61591059	A	0.006	0.383	6.28×10^{-02}
11	rs1000778	FADS3	61655305	A	0.477	-0.080	1.17×10^{-01}
11	rs7942717	FADS3	61647288	G	0.097	-0.080	2.99×10^{-01}
11	rs498793	FADS2	61624705	T	0.313	0.036	4.51×10^{-01}
11	rs17764288	FADS2	61633736	A	0.003	-0.146	4.99×10^{-01}
11	rs482548	FADS2	61633182	T	0.030	-0.010	8.89×10^{-01}

RWAS: Region-wide association study. The region in chromosome 11 containing the FADS1, FADS2 and FADS3 genes was analyzed. Chr: Chromosome. SNP Single Nucleotide Polymorphism. [1]: General linear model (GLM) adjusted for sex, age and diabetes: SNPs were tested in an additive model (0, 1, or 2 minor alleles). Beta: Indicates the regression coefficients per one minor allele. MAF: Minor allele frequency. [2]: Base position in the chromosome (Homo Sapiens GRCh37.p13 genome build used in Illumina HumanOmniExpress-24 BeadChip).

Supplemental Table 8. SNPs in the RWAS for the FADS1/FADS2/FADS3 region in determining serum docosahexaenoic fatty acid concentrations (%) in the whole population.

				Adjusted[1]			
Chr	SNP	Gene	Position[2]	Alleles	MAF	Beta	p
11	rs174547	FADS1	61570783	C	0.298	-0.122	$6.38x10^{-12}$
11	rs174550	FADS1	61571478	C	0.298	-0.122	$7.44x10^{-12}$
11	rs174546	FADS1	61569830	T	0.298	-0.122	$8.22x10^{-12}$
11	rs1535	FADS2	61597972	G	0.322	-0.118	$1.46x10^{-11}$
11	rs174583	FADS2	61609750	T	0.369	-0.115	$1.72x10^{-11}$
11	rs174576	FADS2	61603510	A	0.363	-0.115	$2.24x10^{-11}$
11	rs174577	FADS2	61604814	A	0.392	-0.110	$1.79x10^{-10}$
11	rs2727270	FADS2	61603237	T	0.163	-0.126	$1.49x10^{-05}$
11	rs174570	FADS2	61597212	T	0.228	-0.103	$5.97x10^{-05}$
11	rs2851682	FADS2	61616012	G	0.149	-0.111	$6.03x10^{-04}$
11	rs174611	FADS2	61627881	C	0.116	-0.060	$2.80x10^{-03}$
11	rs174618	FADS2	61629322	C	0.349	-0.039	$2.50x10^{-02}$
11	rs174450	FADS3	61641542	T	0.435	-0.034	$5.20x10^{-02}$
11	rs174455	FADS3	61656117	A	0.407	-0.031	$7.52x10^{-02}$
11	rs12807005	FADS2	61591059	A	0.006	0.122	$1.07x10^{-01}$
11	rs17156442	FADS2	61614023	T	0.098	-0.052	$1.69x10^{-01}$
11	rs498793	FADS2	61624705	T	0.313	0.020	$2.48x10^{-01}$
11	rs1000778	FADS3	61655305	A	0.477	-0.019	$3.20x10^{-01}$
11	rs7942717	FADS3	61647288	G	0.097	-0.014	$6.32x10^{-01}$
11	rs17764288	FADS2	61633736	A	0.003	-0.032	$6.86x10^{-01}$
11	rs482548	FADS2	61633182	T	0.030	0.000	$9.97x10^{-01}$

RWAS: Region-wide association study. The region in chromosome 11 containing the FADS1, FADS2 and FADS3 genes was analyzed. Chr: Chromosome. SNP Single Nucleotide Polymorphism. [1]: General linear model (GLM) adjusted for sex, age and diabetes: SNPs were tested in an additive model (0, 1, or 2 minor alleles). Beta: Indicates the regression coefficients per one minor allele for the adjusted general linear model for sex and age. MAF: Minor allele frequency. [2]: Base position in the chromosome (Homo Sapiens GRCh37.p13 genome build used in Illumina HumanOmniExpress-24 BeadChip).

Supplemental Table 9. SNPs in the RWAS for the FADS1/FADS2/FADS3 region in determining serum omega-6 fatty acid concentrations (%) in the whole population.

				Adjusted[1]			
Chr	SNP	Gene	Position[2]	Alleles	MAF	Beta	p
11	rs174618	FADS2	61629322	C	0.349	0.314	$3.71x10^{-02}$
11	rs174611	FADS2	61627881	C	0.116	0.342	$4.70x10^{-02}$
11	rs17156442	FADS2	61614023	T	0.098	0.576	$7.75x10^{-02}$
11	rs174450	FADS3	61641542	T	0.435	0.217	$1.55x10^{-01}$
11	rs174455	FADS3	61656117	A	0.407	0.180	$2.36x10^{-01}$
11	rs174570	FADS2	61597212	T	0.228	-0.211	$3.43x10^{-01}$
11	rs2851682	FADS2	61616012	G	0.149	0.589	$3.67x10^{-01}$
11	rs2727270	FADS2	61603237	T	0.163	-0.235	$4.02x10^{-01}$
11	rs12807005	FADS2	61591059	A	0.006	-0.208	$4.10x10^{-01}$
11	rs1000778	FADS3	61655305	A	0.477	0.121	$4.52x10^{-01}$
11	rs174583	FADS2	61609750	T	0.369	0.086	$5.71x10^{-01}$
11	rs174577	FADS2	61604814	A	0.392	0.119	$6.12x10^{-01}$
11	rs174576	FADS2	61603510	A	0.363	0.074	$6.26x10^{-01}$
11	rs482548	FADS2	61633182	T	0.030	0.063	$6.78x10^{-01}$
11	rs1535	FADS2	61597972	G	0.322	0.033	$8.28x10^{-01}$
11	rs174546	FADS1	61569830	T	0.298	-0.136	$8.42x10^{-01}$
11	rs174550	FADS1	61571478	C	0.298	0.028	$8.60x10^{-01}$
11	rs174547	FADS1	61570783	C	0.298	0.026	$8.65x10^{-01}$
11	rs498793	FADS2	61624705	T	0.313	0.026	$8.71x10^{-01}$
11	rs17764288	FADS2	61633736	A	0.003	0.024	$8.77x10^{-01}$
11	rs7942717	FADS3	61647288	G	0.097	-0.002	$9.92x10^{-01}$

RWAS: Region-wide association study. The region in chromosome 11 containing the FADS1, FADS2 and FADS3 genes was analyzed. Chr: Chromosome. SNP Single Nucleotide Polymorphism. [1]: General linear model (GLM) adjusted for sex, age and diabetes: SNPs were tested in an additive model (0, 1, or 2 minor alleles). Beta: Indicates the regression coefficients per one minor allele. MAF: Minor allele frequency. [2]: Base position in the chromosome (Homo Sapiens GRCh37.p13 genome build used in Illumina HumanOmniExpress-24 BeadChip).

Supplemental Table 10. SNPs in the RWAS for the ELOV2/ELOV5 region in determining serum omega-3 fatty acid concentrations (%) in the whole population.

				Adjusted[1]			
Chr	SNP	Gene	Position[2]	Alleles	MAF	Beta	p[3]
6	rs3756963	ELOVL2	11022154	C	0.258	-0.078	0.164
6	rs1225737	ELOVL2	10982652	C	0.281	-0.065	0.178
6	rs2295602	ELOVL2	11005842	T	0.317	-0.064	0.185
6	rs12195587	ELOVL2	10989942	A	0.054	0.083	0.226
6	rs8523	ELOVL2	10981053	G	0.327	-0.055	0.250
6	rs3734398	ELOVL2	10982973	T	0.448	-0.054	0.262
6	rs17675073	ELOVL2	11008649	A	0.054	0.077	0.264
6	rs2281591	ELOVL2	10990493	G	0.326	-0.059	0.288
6	rs16870906	ELOVL2	11043039	T	0.087	-0.176	0.323
6	rs17606561	ELOVL2	10982359	A	0.182	-0.053	0.356
6	rs16870891	ELOVL2	11017481	T	0.098	0.139	0.360
6	rs209512	ELOVL5	53203577	C	0.199	0.063	0.362
6	rs3798709	ELOVL2	11001276	G	0.252	-0.042	0.507
6	rs911196	ELOVL2	10990751	G	0.252	-0.038	0.543
6	rs209510	ELOVL5	53202035	C	0.388	-0.028	0.547
6	rs3734397	ELOVL2	10982848	G	0.110	-0.028	0.594
6	rs13204015	ELOVL2	11040460	C	0.014	-0.054	0.598
6	rs7755145	ELOVL2	10995180	T	0.058	0.107	0.637
6	rs976081	ELOVL2	11003875	G	0.110	-0.023	0.669
6	rs1150555	ELOVL2	11000517	C	0.025	-0.062	0.688
6	rs2281274	ELOVL5	53143554	C	0.304	-0.018	0.729
6	rs209515	ELOVL5	53205947	A	0.491	0.012	0.794
6	rs209521	ELOVL5	53210355	T	0.077	0.017	0.813
6	rs209511	ELOVL5	53203007	G	0.104	0.015	0.833
6	rs2235722	ELOVL5	53138912	C	0.189	0.011	0.841
6	rs9468304	ELOVL2	11042165	A	0.302	0.011	0.869
6	rs974323	ELOVL5	53159547	T	0.378	0.007	0.880
6	rs9370194	ELOVL5	53175682	T	0.404	0.005	0.918
6	rs761179	ELOVL5	53166836	C	0.403	0.002	0.975
6	rs4562128	ELOVL5	53202606	C	0.405	0.000	0.999
6	rs209512	ELOVL5	53203577	C	0.199	0.063	0.362

RWAS: Region-wide association study. The region in chromosome 6 containing the ELOV2 and ELOV5 genes was analyzed. Chr: Chromosome. SNP Single Nucleotide Polymorphism. [1]: Model 2, general linear model (GLM) adjusted for sex, age and diabetes: SNPs were tested in an additive model (0, 1, or 2 minor alleles). Beta: Indicates the regression coefficients per one minor allele. MAF: Minor allele frequency. [2]: Base position in the chromosome (Homo Sapiens GRCh37.p13 genome build used in Illumina HumanOmniExpress-24 BeadChip).

Supplemental Table 11. SNPs in the RWAS for the ELOV2/ELOV5 region in determining serum omega-6 fatty acid concentrations (%) in the whole population.

						Adjusted[1]	
Chr	SNP	Gene	Position[2]	Alleles	MAF	Beta	p
6	rs2295602	ELOVL2	11005842	T	0.317	0.353	0.021
6	rs8523	ELOVL2	10981053	G	0.327	0.345	0.023
6	rs3734398	ELOVL2	10982973	T	0.448	0.341	0.025
6	rs3734397	ELOVL2	10982848	G	0.110	-0.318	0.058
6	rs1225737	ELOVL2	10982652	C	0.281	0.286	0.061
6	rs3798709	ELOVL2	11001276	G	0.252	0.334	0.093
6	rs911196	ELOVL2	10990751	G	0.252	0.320	0.107
6	rs976081	ELOVL2	11003875	G	0.110	-0.268	0.114
6	rs9468304	ELOVL2	11042165	A	0.302	0.321	0.123
6	rs3756963	ELOVL2	11022154	C	0.258	0.203	0.249
6	rs2281591	ELOVL2	10990493	G	0.326	0.198	0.262
6	rs17606561	ELOVL2	10982359	A	0.182	0.184	0.310
6	rs2235722	ELOVL5	53138912	C	0.189	-0.152	0.369
6	rs1150555	ELOVL2	11000517	C	0.025	-0.354	0.469
6	rs13204015	ELOVL2	11040460	C	0.014	-0.202	0.532
6	rs16870906	ELOVL2	11043039	T	0.087	-0.344	0.541
6	rs209512	ELOVL5	53203577	C	0.199	0.127	0.558
6	rs17675073	ELOVL2	11008649	A	0.054	-0.121	0.579
6	rs209511	ELOVL5	53203007	G	0.104	0.123	0.597
6	rs12195587	ELOVL2	10989942	A	0.054	-0.108	0.621
6	rs4562128	ELOVL5	53202606	C	0.405	-0.039	0.802
6	rs9370194	ELOVL5	53175682	T	0.404	-0.032	0.837
6	rs209521	ELOVL5	53210355	T	0.077	0.046	0.842
6	rs2281274	ELOVL5	53143554	C	0.304	-0.032	0.843
6	rs209510	ELOVL5	53202035	C	0.388	0.029	0.845
6	rs7755145	ELOVL2	10995180	T	0.058	0.114	0.874
6	rs209515	ELOVL5	53205947	A	0.491	-0.019	0.900
6	rs16870891	ELOVL2	11017481	T	0.098	0.052	0.914
6	rs974323	ELOVL5	53159547	T	0.378	-0.016	0.920
6	rs761179	ELOVL5	53166836	C	0.403	-0.012	0.937

RWAS: Region-wide association study. The region in chromosome 6 containing the ELOV2 and ELOV5 genes was analyzed. Chr: Chromosome. SNP Single Nucleotide Polymorphism. [1]: General linear model (GLM) adjusted for sex, age and diabetes: SNPs were tested in an additive model (0, 1, or 2 minor alleles). Beta: Indicates the regression coefficients per one minor allele. MAF: Minor allele frequency. [2]: Base position in the chromosome (Homo Sapiens GRCh37.p13 genome build used in Illumina HumanOmniExpress-24 BeadChip).

Supplemental Table 12. SNPs in the RWAS for the ELOV2/ELOV5 region in determining linoleic fatty acid concentrations (%) in the whole population.

					Adjusted[1]		
Chr	SNP	Gene	Position[2]	Alleles	MAF	Beta	p
6	rs8523	ELOVL2	10981053	G	0.327	0.390	0.024
6	rs2295602	ELOVL2	11005842	T	0.317	0.390	0.024
6	rs3734398	ELOVL2	10982973	T	0.448	0.381	0.026
6	rs1225737	ELOVL2	10982652	C	0.281	0.368	0.032
6	rs3798709	ELOVL2	11001276	G	0.252	0.428	0.057
6	rs911196	ELOVL2	10990751	G	0.252	0.404	0.072
6	rs3734397	ELOVL2	10982848	G	0.110	-0.293	0.122
6	rs9468304	ELOVL2	11042165	A	0.302	0.355	0.132
6	rs2235722	ELOVL5	53138912	C	0.189	-0.262	0.170
6	rs17675073	ELOVL2	11008649	A	0.054	-0.326	0.187
6	rs976081	ELOVL2	11003875	G	0.110	-0.237	0.217
6	rs12195587	ELOVL2	10989942	A	0.054	-0.298	0.227
6	rs2281274	ELOVL5	53143554	C	0.304	0.191	0.294
6	rs13204015	ELOVL2	11040460	C	0.014	-0.341	0.351
6	rs2281591	ELOVL2	10990493	G	0.326	0.174	0.384
6	rs3756963	ELOVL2	11022154	C	0.258	0.168	0.398
6	rs16870891	ELOVL2	11017481	T	0.098	0.435	0.420
6	rs17606561	ELOVL2	10982359	A	0.182	0.143	0.485
6	rs209511	ELOVL5	53203007	G	0.104	0.175	0.505
6	rs4562128	ELOVL5	53202606	C	0.405	-0.112	0.527
6	rs9370194	ELOVL5	53175682	T	0.404	-0.088	0.613
6	rs209521	ELOVL5	53210355	T	0.077	0.120	0.649
6	rs974323	ELOVL5	53159547	T	0.378	-0.074	0.673
6	rs761179	ELOVL5	53166836	C	0.403	-0.069	0.694
6	rs7755145	ELOVL2	10995180	T	0.058	0.318	0.695
6	rs209515	ELOVL5	53205947	A	0.491	-0.061	0.716
6	rs16870906	ELOVL2	11043039	T	0.087	0.190	0.765
6	rs209510	ELOVL5	53202035	C	0.388	0.030	0.856
6	rs209512	ELOVL5	53203577	C	0.199	-0.032	0.898
6	rs1150555	ELOVL2	11000517	C	0.025	0.042	0.940

RWAS: Region-wide association study. The region in chromosome 6 containing the ELOV2 and ELOV5 genes was analyzed. Chr: Chromosome. SNP Single Nucleotide Polymorphism. [1]: Model 2, general linear model (GLM) adjusted for sex, age and diabetes: SNPs were tested in an additive model (0, 1, or 2 minor alleles). Beta: Indicates the regression coefficients per one minor allele. MAF: Minor allele frequency. [2]: Base position in the chromosome (Homo Sapiens GRCh37.p13 genome build used in Illumina HumanOmniExpress-24 BeadChip).

Supplemental Figure 10. Serum omega-6/omega-3 PUFA ratio depending on the FADS1-rs174547 genotype in the whole population. Genotype prevalence was: TT 49.3% (n=212), TC 41.0% (n=172) and CC 9.7% (n=42). The minor allele (C) was associated with higher concentrations in all the adjusted models. 1: Unadjusted model; 2: Model adjusted for sex, age and diabetes; 3: Model adjusted for sex, age, diabetes, body mass index, medications (antihypertensive, hypolipidemic and antidiabetic drugs), smoking, physical activity and adherence to Mediterranean diet. Values are means by genotype.

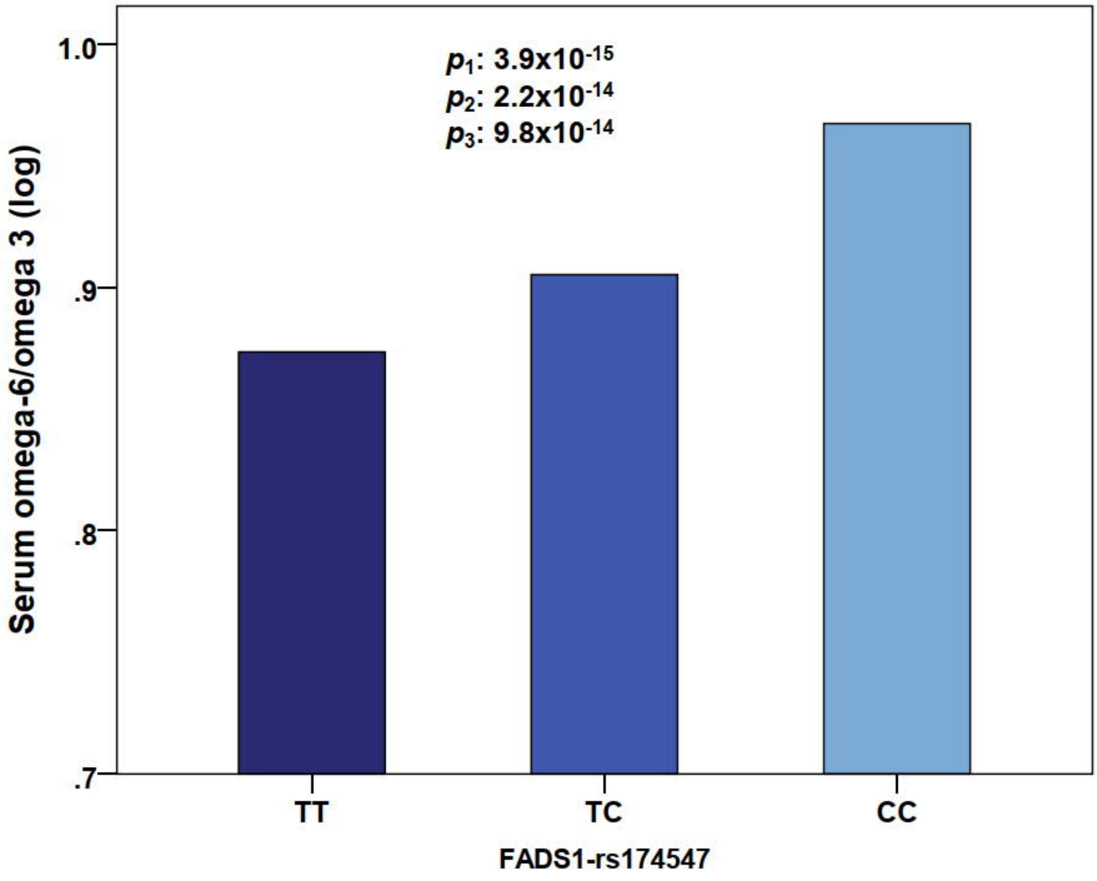

Supplemental Figure 11. Manhattan plot for the GWAs interaction results between the top-ranked SNPs and the adherence to the Mediterranean diet in determining serum omega-6/omega-3 PUFA ratio in the whole population. Associations were obtained from the genetic additive model and *p*-values were expressed as $-\log_{10}$(*p*-value). The red line represents the threshold for suggestive GWAS significance ($-\log_{10}(1 \times 10^{-5})$).

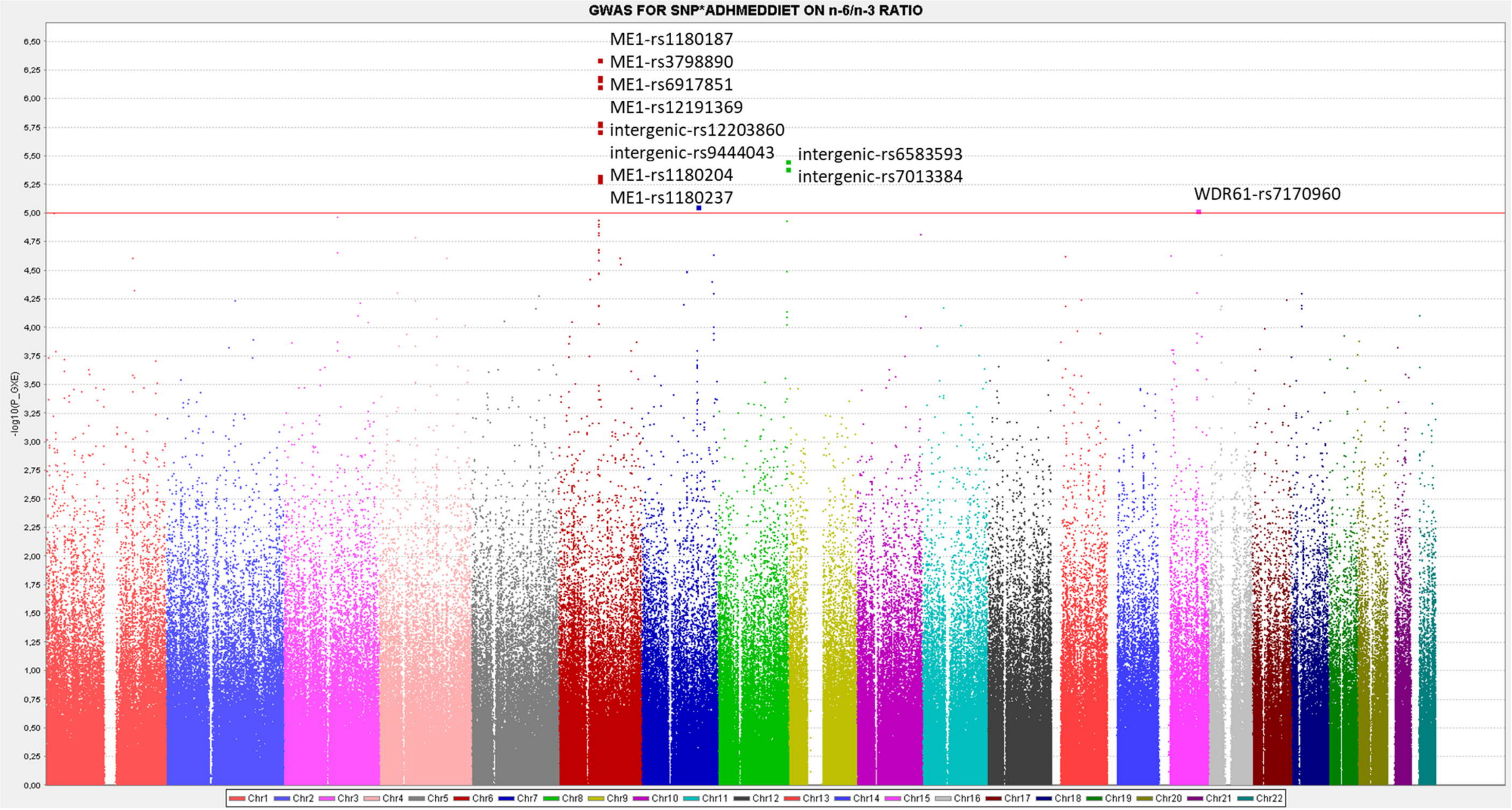

References

In this section reference items are following the same order as the References section in the main document.

1. Chilton, F.; Dutta, R.; Reynolds, L.; Sergeant, S.; Mathias, R.; Seeds, M. Precision Nutrition and Omega-3 Polyunsaturated Fatty Acids: A Case for Personalized Supplementation Approaches for the Prevention and Management of Human Diseases. Nutrients 2017, 9, 1165.
2. Park, H.G.; Park, W.J.; Kothapalli, K.S.D.; Brenna, J.T. The fatty acid desaturase 2 (FADS2) gene product catalyzes Δ4 desaturation to yield n-3 docosahexaenoic acid and n-6 docosapentaenoic acid in human cells. The FASEB Journal 2015, 29, 3911–3919. Ç
3. Kim, O.; Lee, S.; An, W. Impact of Blood or Erythrocyte Membrane Fatty Acids for Disease Risk Prediction: Focusing on Cardiovascular Disease and Chronic Kidney Disease. Nutrients 2018, 10, 1454.
